# Multitask Deep Learning Model for Predicting Lymph Node Metastasis and Disease‐Free Survival From Cytology Images in Bladder Cancer: A Multicenter Study With Pan‐Cytology Validation

**DOI:** 10.1002/advs.76577

**Published:** 2026-10-21

**Authors:** Guibin Hong, Fan Jiang, Ye Xie, Xiaowei Huang, Runnan Shen, Yun Wang, Mengyi Zhu, Haoxuan Wang, Hongkun Yang, Yanxiao Nie, Zhen Lin, Rui Chen, Nengtai Ouyang, Tianxin Lin, Shaoxu Wu

**Affiliations:** ^1^ Department of Urology Sun Yat‐sen Memorial Hospital of Sun Yat‐sen University Guangzhou China; ^2^ Guangdong Provincial Clinical Research Centre for Urological Diseases Guangdong China; ^3^ CellsVision Medical Technology Services Co., Ltd Guangzhou China; ^4^ Department of Pathology Sun Yat‐sen Memorial Hospital of Sun Yat‐sen University Guangzhou China; ^5^ Guangdong Provincial Key Laboratory of Malignant Tumour Epigenetics and Gene Regulation Guangdong‐Hong Kong Joint Laboratory for RNA Medicine Sun Yat‐sen Memorial Hospital of Sun Yat‐sen University Guangzhou China

**Keywords:** area under the curve, bladder cancer, cervical cancer, cohort, cytology, cytopathology, internal medicine, medicine, oncology, thyroid cancer

## Abstract

Accurate prediction of lymph node metastasis (LNM) and survival outcomes is crucial for personalized treatment in bladder cancer, yet remains clinically challenging. This study aimed to develop a Cytology‐based multitask predictive Network (CytoNet) to simultaneously predict LNM and disease‐free survival (DFS) in bladder cancer, and to evaluate its generalizability in cervical and thyroid cancers. This study included a multicenter cohort of 5273 patients, with bladder cancer (n = 1134), cervical cancer (n = 598), and thyroid cancer (n = 3541). CytoNet was constructed through a three‐stage pipeline comprising self‐supervised pretraining, supervised fine‐tuning, and multitask prediction. In bladder cancer, CytoNet achieved an area under the curve (AUC) of 0.831‐0.881 for LNM prediction and a C index of 0.680‐0.710 for DFS prediction across all cohorts, outperforming single‐task models and standard radiological assessments. In cervical cancer, CytoNet demonstrated robust performance for both LNM prediction (AUC 0.847‐0.862) and DFS prediction (C index 0.690‐0.709), while in thyroid cancer, it achieved an AUC of 0.819‐0.855 for LNM prediction. In conclusion, CytoNet represents a reliable tool for predicting LNM and prognosis from cytopathology images across multiple cancer types, showing potential as a valuable adjunct to current clinical decision‐making. Further prospective validation is warranted to assess its clinical utility.

## Graphical abstract

Through self‐supervised pretraining and supervised fine‐tuning on large‐scale cytology image datasets, CytoNet predicts lymph node metastasis and survival. Across independent cohorts, this multitask framework demonstrates robust performance, improves risk stratification beyond radiological assessment, and provides interpretable evidence to support individualized clinical decision‐making.

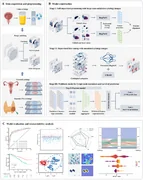

## 1 Introduction

Bladder cancer is the ninth most common cancer globally and represents one of the most prevalent cancers of the urinary system [1]. Despite advancements in multimodal therapies, the rates of recurrence and metastasis remain high [2, 3]. Lymph node metastasis (LNM) is the most common pathway of metastasis for bladder cancer, and the prognosis of patients with nodal involvement is significantly worse [4, 5, 6]. Therefore, accurate prediction of LNM is critical for optimizing personalized treatment decisions and has the potential to improve patient outcomes.

Currently, preoperative assessment of LNM relies on imaging examinations such as computed tomography (CT) and magnetic resonance imaging (MRI) [7, 8, 9]. However, these methods have limited sensitivity and specificity for detecting micrometastases or differentiating reactive lymph node enlargement, potentially leading to undertreatment or overtreatment [10, 11]. Since definitive diagnosis still depends on postoperative pathological diagnosis, this delay constrains optimal preoperative planning. Thus, there is an urgent need for a noninvasive and precise tool capable of predicting LNM risk preoperatively.

Deep learning has shown great promise in medical image analysis by extracting complex morphological features that are difficult to discern by humans [12, 13, 14, 15]. Several studies have demonstrated the potential of deep learning‐based models for predicting LNM and prognosis in various cancers [16, 17, 18, 19, 20, 21]. Notably, one study has shown that a deep learning model based on cytology images can effectively predict central LNM in papillary thyroid microcarcinoma, and another study demonstrates that a cytology‐based deep learning model can accurately identify the tumor origin in cancers of unknown primary [22, 23]. These findings collectively underscore the value of cytology images in predicting tumor aggressiveness and guiding clinical management. Urine cytology, a noninvasive, cost‐effective, and routinely used method for diagnosis and follow‐up, allows for the direct collection of exfoliated tumor cells from the urinary tract [9, 24]. These cells not only indicate the presence of malignancy, but their morphological features and spatial architecture may also contain information reflecting tumor invasiveness [25, 26, 27, 28]. Prior studies have explored the application of deep learning in urine cytology images to assist in bladder cancer screening or staging [26, 29]. However, existing cytology‐based models have primarily focused on cancer detection or local tumor staging, and the potential of cytology images to provide noninvasive information on metastatic risk and long‐term prognosis remains insufficiently explored. We hypothesized that the morphology and spatial organization of exfoliated tumor cells may reflect tumor aggressiveness beyond conventional cytological diagnosis and could be leveraged for LNM prediction, prognostic assessment, and treatment‐relevant risk stratification.

Traditionally, deep learning models used for bladder cancer have mostly been designed to perform a single task, failing to fully utilize the complementary information among different tasks. Multitask deep learning, by simultaneously optimizing multiple related tasks within a unified architecture and leveraging shared feature representations, can effectively enhance data utilization efficiency and model generalizability [30, 31, 32, 33, 34]. It has been proven to outperform single‐task models in medical applications [35].

In this study, a deep learning model, called Cytology‐based multitask predictive Network (CytoNet), was developed and validated utilizing cytology images to simultaneously predict LNM and disease‐free survival (DFS) in a multicenter cohort of patients with bladder cancer. To further validate the CytoNet, we applied it to cervical and thyroid cancers, which also rely on cytological examinations, to test its generalizability.

## 2 Methods

### 2.1 Study Design and Patient Recruitment

In this retrospective, multicenter study, we included patients with bladder cancer, treated with radical cystectomy and lymph node dissection, from four Sun Yat‐sen University‐affiliated medical centers. The inclusion and exclusion criteria were detailed in Supplementary Material (p 3). Finally, 1032 patients were eligible and included. Of these, patients from Sun Yat‐sen Memorial Hospital of Sun Yat‐sen University (SYSMH, between January 14, 2014, and March 30, 2025) were stratified by LNM status and randomly allocated to the training cohort (TC, n = 478) and internal validation cohort (IVC, n = 205) at a ratio of 7:3. Patients from the Third Affiliated Hospital of Sun Yat‐sen University (between January 9, 2014, and June 25, 2025) were assigned to external validation cohort 1 (EVC1, n = 276). Patients from the Fifth Affiliated Hospital of Sun Yat‐sen University (between February 20, 2015, and June 30, 2025) and Shenshan Central Hospital (between March 15, 2022, and June 22, 2025) were assigned to external validation cohort 2 (EVC2, n = 73). The TC, IVC, EVC1, and EVC2 were collectively referred to as the combined cohort, whereas the IVC, EVC1, and EVC2 were referred to as the combined validation cohort. The sample size calculation was described in Supplementary Material (pp 3‐4).

Ethics approval was obtained from the ethics committee of all participating hospitals, and the requirement for informed consent was waived because of the retrospective nature of the study.

### 2.2 Data Collection and Image Digitization

Baseline clinical and pathological data were collected from institutional medical records, including patient age, sex, imaging characteristics (tumor size, tumor number, lymph node size, clinical tumor and lymph node stage), pathological tumor and lymph node stage, American Joint Committee on Cancer (AJCC) stage, and whether postoperative adjuvant therapy was used. Postoperative histopathological lymph node status was used as the reference standard for LNM in model development and validation. Data on race and ethnicity were not collected because these variables are not routinely recorded in the institutional medical records of the participating hospitals. Follow‐up information was obtained through comprehensive reviews of medical records, imaging databases, and telephone interviews. DFS was defined as the time from surgery to the first documented disease recurrence, distant metastasis, death from any cause, or the last follow‐up. Overall survival (OS) was defined as the time from surgery to death from any cause or the last follow‐up. Liquid‐based cytology slides prepared using Papanicolaou staining were retrieved from pathology archives and digitized as whole slide images (WSIs) at 40× magnification using four types of high‐resolution scanners (KF‐PRO‐400, Ningbo, China; Pannoramic SCAN II, Budapest, Hungary; SQS‐600P, Shenzhen, China; PRECICE 600, Beijing, China). No scanner‐specific model retraining, scanner‐specific normalization, or scanner‐specific threshold adjustment was applied. Instead, all WSIs acquired from different scanners underwent the same patch extraction and model inference workflow. Two cytopathologists reviewed all the WSIs to ensure image quality and confirm the presence of tumor cells.

### 2.3 Development of the CytoNet

We developed a multitask deep learning framework, named CytoNet, to simultaneously predict LNM and DFS from cytology images. The rationale for combining these tasks in a unifying model lies in their inherent clinical and biological interconnection: LNM represents the primary pathway of metastasis in bladder cancer and is strongly associated with poor prognosis, while DFS serves as a comprehensive indicator of disease progression and treatment outcome. By leveraging shared representations across these related tasks, the multitask approach enhances feature learning and improves model generalizability. The CytoNet was developed through a multi‐stage pipeline, as illustrated in Figure 1. In the first stage, self‐supervised pretraining was performed using the DINO framework to learn generalizable visual representations of cellular morphology. The RegNetY backbone was pretrained on a large‐scale dataset of over 100 000 unlabeled cytology images derived from diverse origins, including urine, cervical specimens, and thyroid fine‐needle aspirations. The second stage involved supervised fine‐tuning to specialize the feature extractor for urine cytology images. The pretrained RegNetY was enhanced with a Convolutional Block Attention Module (CBAM) and fine‐tuned using patch‐level annotations across six detailed cytological categories, thereby refining its ability to discriminate morphologically relevant features. The third stage introduced a Top‐N Feature (TNF) model for multitask prediction. The most salient image patches were selected and aggregated into a slide‐level feature representation using an attention‐based mechanism. The resulting features are processed by two dedicated output branches for LNM and DFS predictions, which are jointly optimized under a weighted multitask loss function. To mitigate class imbalance in the LNM prediction task, LNM‐positive samples were oversampled during model training to increase their representation in training batches, whereas the validation and test cohorts were kept unchanged to reflect the original disease prevalence.

**FIGURE 1:**
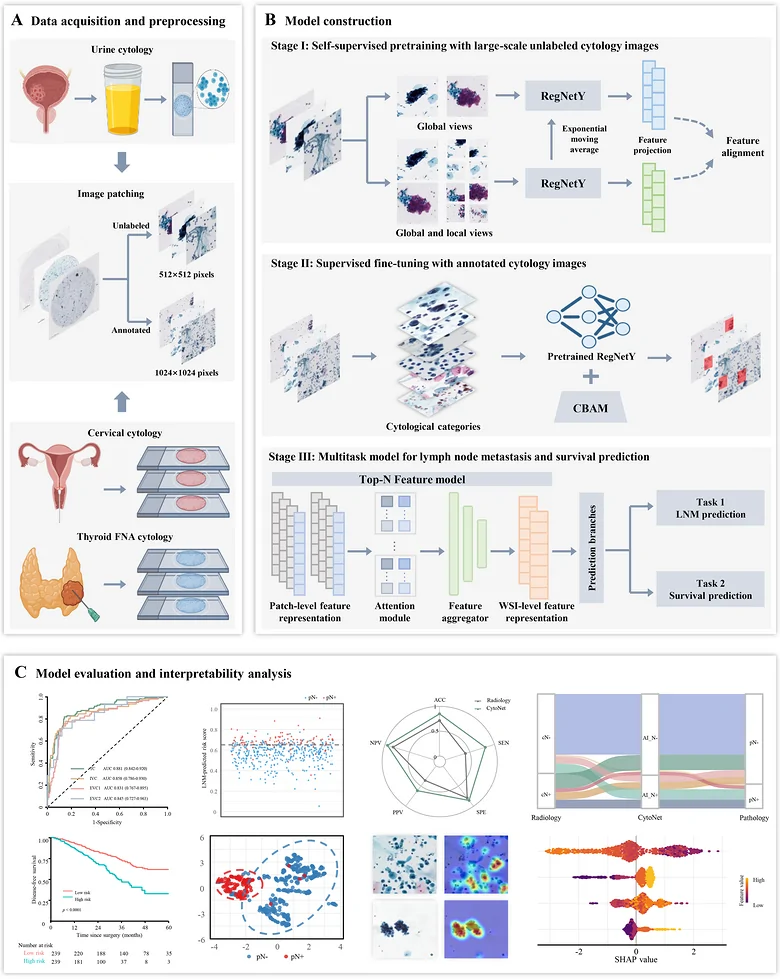
Schematic diagram of the study workflow. (A) Data acquisition and preprocessing. Cytology slides were acquired and processed to generate image patches. (B) Model construction. The CytoNet was developed through a multi‐stage pipeline, which involved self‐supervised pretraining, supervised fine‐tuning, and multitask prediction. (C) Model evaluation and interpretability analysis. CytoNet, Cytology‐based multitask predictive Network; CBAM, Convolutional Block Attention Module; LNM, lymph node metastasis.

To enhance the interpretability of CytoNet, we employed multiple techniques to elucidate its decision‐making process. First, the high‐dimensional features extracted from the layer preceding the final prediction branch of the TNF module were visualized using Uniform Manifold Approximation and Projection (UMAP), revealing the intrinsic structure and separability of learned representations in the feature space. Furthermore, to identify the specific cytological regions that most critically influenced the CytoNet's prediction, we applied Class Activation Mapping (CAM). This technique generated visual heatmaps overlaid on the original urine cytology images, highlighting salient cellular and morphological areas that served as the primary drivers of the model's output. The integrated framework provides an end‐to‐end analytical pipeline from cellular morphology to clinical outcomes. Further implementation details are provided in Supplementary Material (pp 4‐9).

### 2.4 Evaluation of the CytoNet for Predicting LNM

The performance of CytoNet for LNM prediction was assessed using receiver operating characteristic analysis, with the area under the curve (AUC) as the primary metric. Patients were stratified​ into low‐risk and high‐risk groups based on the LNM‐predicted risk scores, using the optimal cutoff determined by the Youden index in the TC. To evaluate the CytoNet's clinical utility, the calibration curve was plotted to visualize the consistency between the model's predicted probabilities and actual observed probabilities, and the decision curve analysis was performed to quantify the net benefit of the model across different threshold probabilities.

To benchmark the deep learning model against conventional diagnostic methods, we compared the LNM predictions of CytoNet with those from preoperative CT or MRI imaging. Radiology‐based LNM status was extracted from the original preoperative CT or MRI reports issued before surgery. Cases were classified as radiologically LNM‐positive when the report described suspicious or metastatic lymph nodes, and as radiologically LNM‐negative when no suspicious lymph node metastasis was reported. The net reclassification improvement (NRI) was computed to evaluate the improvement in classification accuracy, and the Sankey diagram was employed to visually depict the reclassification pathways. Furthermore, significant clinical predictors associated with LNM were identified using Least Absolute Shrinkage and Selection Operator (LASSO) regression in the TC and used to construct a clinical model. An integrated model was built by incorporating both the selected clinical predictors and the LNM‐predicted risk scores generated by CytoNet. The performance of CytoNet, the clinical model, and the integrated model was evaluated and compared. Furthermore, to quantify the contribution of individual input variables for model prediction, we calculated SHapley Additive exPlanations (SHAP) values.

To further evaluate whether CytoNet could provide complementary information for current preoperative LNM assessment, we conducted an exploratory blinded reader study. A total of 150 patients were randomly selected from the validation cohorts, and no patients from the training cohort were included. Six radiologists, including three junior and three senior radiologists, independently reviewed routinely available clinical information and preoperative CT or MRI images, and provided a judgment of LNM status. All readers were blinded to postoperative pathological diagnoses, CytoNet predictions, and the assessments of other readers. For the same patients, CytoNet generated LNM‐predicted risk scores based on urine cytology WSIs. After a 3‐week washout period, the radiologists reassessed the cases in a randomized order using the same clinical and radiological information, with additional access to cytology images, CAM heatmaps, and CytoNet‐derived LNM‐predicted risk scores. The performance of radiologists alone, CytoNet alone, and CytoNet‐assisted radiologists was compared using postoperative pathological LNM status as the reference standard.

### 2.5 Evaluation of the CytoNet for Predicting Prognosis

The prognostic performance of CytoNet was evaluated using Harrell's concordance index (C index). Based on the median value of the predicted survival probability in the TC, patients were stratified into high‐risk and low‐risk groups. This cutoff was subsequently applied to all validation cohorts. Univariable and multivariable Cox proportional hazards regression analyses were conducted to identify significant clinical factors associated with DFS. These selected factors were used to build a clinical Cox model. An integrated Cox model was then developed by incorporating both the significant clinical factors and the survival probabilities generated by CytoNet.

In a post‐hoc analysis, we investigated the ability of CytoNet to predict the benefit of adjuvant therapy. Leveraging its dual‐task nature, patients were categorized into four distinct groups: class 1 (low LNM risk and high survival score), class 2 (low LNM risk and low survival score), class 3 (high LNM risk and high survival score), and class 4 (high LNM risk and low survival score). We subsequently performed interaction analyses within the AJCC stage III subgroup to test whether the effect of adjuvant therapy on survival outcomes differed significantly across these predefined risk classes. Furthermore, we compared the performance of CytoNet against the single‐task model.

### 2.6 Independent External Testing in Bladder Cancer

To further evaluate the transportability of CytoNet across institutions outside the Sun Yat‐sen University‐affiliated hospital system, we included an independent external test cohort (IETC, Table S1) comprising 102 patients from three centers: Nanfang Hospital of Southern Medical University (n = 49, March 2020 to September 2025), Guangzhou First People's Hospital (n = 32, November 2021 to September 2025), and the First Affiliated Hospital of Shantou University Medical College (n = 21, January 2022 to October 2025). The locked CytoNet was directly applied to the IETC without retraining, hyperparameter tuning, or threshold adjustment. This cohort was analyzed separately from the Sun Yat‐sen University‐affiliated medical centers and was used for independent evaluation of model transportability.

### 2.7 Pan‐Cytology Cohorts

To evaluate the generalizability of the CytoNet, we further validated it on​ cervical and thyroid cancer cohorts. These two cancer types were selected because they similarly rely on cytological examination. For pan‐cytology validation, the same DINO‐pretrained RegNetY backbone was used as the shared initial feature extractor across cancer types. However, supervised fine‐tuning and TNF multitask model training were performed separately within each cancer‐specific cohort. The generalizability assessed in this study refers to the transferability of a unified cytology‐based modeling framework and shared self‐supervised representations, rather than a single universally fine‐tuned model applied across all cancer types without adaptation. For cervical cancer, we included 598 eligible patients from SYSMH who had undergone radical hysterectomy and lymph node dissection. These patients were divided into a cervical training cohort (C‐TC, n = 423) and a cervical validation cohort (C‐VC, n = 175) for the prediction of both LNM and DFS. For thyroid cancer, 3541 eligible patients from SYSMH treated with thyroidectomy and lymph node dissection were included. These patients were divided into a thyroid training cohort (T‐TC, n = 2514) and a thyroid validation cohort (T‐VC, n = 1027) for the prediction of LNM. The detailed patient selection workflow for both cancers is provided in Figure S1.

### 2.8 Statistical Analysis

All statistical analyses were performed using R software (version 4.4.1). Continuous variables were analyzed using the t‐test or Mann‐Whitney U test, and categorical variables were analyzed using the χ^2^ test or Fisher's exact test. Performance metrics for LNM prediction, including accuracy, sensitivity, specificity, positive predictive value, and negative predictive value, were calculated along with their 95% confidence intervals (CIs). The DeLong test was used to compare AUCs between different models evaluated on the same dataset. Survival outcomes between risk groups were compared using Kaplan–Meier curves and the log‐rank test. The Bonferroni method was applied to adjust *p*‐values for multiple comparisons where applicable. Statistical tests were two‐sided, with *p* < 0.05 considered statistically significant. Patients with missing data required for model development or evaluation, including cytology images, preoperative imaging, postoperative pathological lymph node status, or follow‐up information, were excluded during eligibility screening. No statistical imputation was performed. For survival analyses, patients without documented recurrence, metastasis, or death were censored at the date of last follow‐up.

## 3 Results

### 3.1 Participant Characteristics

The detailed clinicopathologic characteristics of the 1134 patients with bladder cancer across the five cohorts are summarized in Table S1. Among them, 975 (86.0%) patients were male, with a median age of 66 years (interquartile range [IQR] 59‐73). The prevalence of pathologically confirmed LNM was comparable across five cohorts, ranging from 19.2% to 22.5%. The median follow‐up for DFS ranged from 20 to 31 months, and for OS ranged from 22 to 35 months. The patient characteristics for cervical and thyroid cancer are detailed in Table S2.​ In the cervical cancer cohort, the median age was 52 years (IQR 45‐58). The rates of pathologically confirmed LNM were 22.0% in the C‐TC and 22.3% in the C‐VC. The median follow‐up was 37 months for DFS and 38 months for OS. For the thyroid cancer cohort, the median age was 42 years (IQR 33‐52), and female patients constituted 76.1% (n = 2693). The prevalence of LNM were 52.2% in the T‐TC and 53.8% in the T‐VC. Follow‐up data were not available for the thyroid cancer cohort.

### 3.2 Performance of the Model for Predicting LNM in Bladder Cancer

The CytoNet achieved AUCs of 0.881 (95% CI 0.842‐0.920) in TC, 0.858 (0.786‐0.930) in IVC, 0.831 (0.767‐0.895) in EVC1, and 0.845 (0.727‐0.963) in EVC2, respectively (Figure 2A). Calibration curves demonstrated good agreement between the predicted probabilities of LNM and the observed outcomes across the TC, IVC, EVC1, and EVC2 (Figure 2B, Table S3). Decision curve analyses indicated that the clinical utility of CytoNet was favorable across a wide range of threshold probabilities (Figure 2C). The model‐derived LNM risk scores showed a clear separation between patients with and without LNM (Figure 2D). In the comprehensive comparison, CytoNet demonstrated superior performance to standard radiological assessment across multiple metrics (Figure 2E, Table 1). This improvement was quantified by a significant NRI when CytoNet predictions were compared to radiological assessment (Figure 2F). The CytoNet maintained higher predictive accuracy than radiology for both node‐negative and node‐positive subgroups (Figure 2G), as detailed in the confusion matrices (Figure 2H). The Sankey diagram visualized the concordance between radiological assessment, CytoNet prediction, and the final pathological diagnosis, underscoring the model's alignment with ground truth (Figure 2I and Figure S2). In an analysis across different digital scanners, the CytoNet demonstrated robust predictive performance for LNM, with AUCs ranging from 0.814 to 0.911 (Figure S3). In the subgroup of patients who received neoadjuvant therapy, the CytoNet achieved superior comprehensive performance compared to standard radiology assessment (Figure S4). We also compared the CytoNet with the single‐task model for LNM prediction. The CytoNet demonstrated superior performance, with AUCs improving to a range of 0.831–0.881 from 0.776–0.825 achieved by the single‐task model (Figure 2J).

**FIGURE 2:**
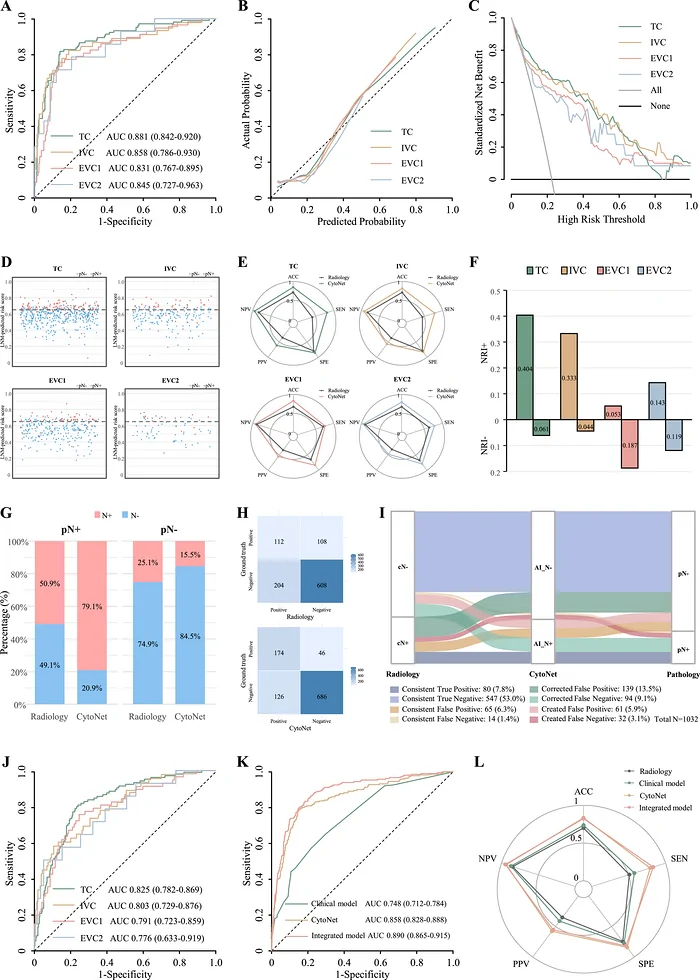
Model performance for predicting lymph node metastasis in bladder cancer. (A–C) Receiver operating characteristic curves, calibration curves, and decision curve analysis curves evaluate the predictive accuracy and clinical utility of CytoNet across different cohorts. (D) Scatter plots demonstrate the distribution of LNM‐predicted risk scores. (E) Radars charts display the comparison of multiple performance metrics between the CytoNet and radiology. (F) Net reclassification improvement analyses show the performance change in predicting lymph node metastasis of the CytoNet compared with radiology. (G) Bar charts compare the prediction performance of radiology and CytoNet across different lymph node statuses in the combined cohort. (H) Confusion matrices compare the performance of the CytoNet and radiology. (I) Sankey diagram illustrating the concordance and reclassification relationships among radiological assessment, CytoNet prediction, and postoperative pathological lymph node status in the combined cohort. The left, middle, and right columns represent radiology‐based nodal assessment, CytoNet‐predicted nodal status, and pathological reference status, respectively. cN‐/cN+ indicate radiology‐based negative or positive lymph node status; AI_N‐/AI_N+ indicate CytoNet‐predicted negative or positive lymph node status; and pN‐/pN+ indicate pathologically negative or positive lymph node status. The width of each flow is proportional to the number of patients. “Consistent” indicates that radiology and CytoNet gave the same classification relative to pathology; “corrected” indicates that CytoNet corrected a radiology misclassification; and “created” indicates that CytoNet introduced a new misclassification relative to radiology. (J) Receiver operating characteristic curves of the single‐task model across different cohorts. (K) Receiver operating characteristic curves compare the predictive performance of the clinical model, CytoNet, and the integrated model in the combined cohort. (L) The radar chart displays the comparison among radiology, the clinical model, CytoNet, and the integrated model. CytoNet, Cytology‐based multitask predictive Network; TC, training cohort; IVC, internal validation cohort; EVC1, external validation cohort 1; EVC2, external validation cohort 2; AUC, area under the curve; ACC, accuracy; SEN, sensitivity; SPE, specificity; PPV, positive predictive value; NPV, negative predictive value; NRI, net reclassification improvement.

**TABLE 1:** Performance of the CytoNet and radiology in predicting lymph node metastasis.

	ACC (95% CI)	SEN (95% CI)	SPE (95% CI)	PPV (95% CI)	NPV (95% CI)
**CytoNet**					
TC	0.849 (0.815‐0.879)	0.817 (0.732‐0.880)	0.858 (0.819‐0.890)	0.616 (0.533‐0.693)	0.944 (0.914‐0.964)
IVC	0.820 (0.761‐0.866)	0.778 (0.637‐0.875)	0.831 (0.766‐0.881)	0.565 (0.441‐0.681)	0.930 (0.876‐0.962)
EVC1	0.826 (0.777‐0.866)	0.754 (0.629‐0.848)	0.845 (0.791‐0.887)	0.558 (0.447‐0.664)	0.930 (0.885‐0.958)
EVC2	0.795 (0.688‐0.871)	0.786 (0.524‐0.924)	0.797 (0.677‐0.880)	0.478 (0.292‐0.670)	0.940 (0.838‐0.979)
C‐TC	0.825 (0.786‐0.858)	0.763 (0.668‐0.838)	0.842 (0.799‐0.878)	0.577 (0.489‐0.661)	0.927 (0.891‐0.951)
C‐VC	0.811 (0.747‐0.863)	0.744 (0.589‐0.854)	0.831 (0.759‐0.885)	0.558 (0.423‐0.684)	0.919 (0.857‐0.955)
T‐TC	0.790 (0.774‐0.805)	0.783 (0.760‐0.804)	0.798 (0.774‐0.819)	0.809 (0.786‐0.830)	0.771 (0.747‐0.793)
T‐VC	0.774 (0.748‐0.799)	0.763 (0.726‐0.797)	0.787 (0.748‐0.821)	0.807 (0.771‐0.838)	0.740 (0.700‐0.776)
**Radiology**					
TC	0.713 (0.671‐0.752)	0.413 (0.324‐0.509)	0.797 (0.753‐0.835)	0.361 (0.281‐0.451)	0.830 (0.788‐0.865)
IVC	0.712 (0.647‐0.770)	0.444 (0.309‐0.588)	0.787 (0.718‐0.844)	0.370 (0.254‐0.504)	0.834 (0.767‐0.885)
EVC1	0.667 (0.609‐0.720)	0.702 (0.573‐0.805)	0.658 (0.593‐0.717)	0.348 (0.267‐0.438)	0.894 (0.837‐0.933)
EVC2	0.671 (0.557‐0.768)	0.643 (0.388‐0.837)	0.678 (0.551‐0.783)	0.321 (0.179‐0.507)	0.889 (0.765‐0.952)
C‐TC	0.657 (0.611‐0.701)	0.495 (0.395‐0.594)	0.703 (0.652‐0.750)	0.319 (0.249‐0.399)	0.832 (0.783‐0.871)
C‐VC	0.651 (0.578‐0.718)	0.538 (0.386‐0.684)	0.684 (0.602‐0.756)	0.328 (0.226‐0.450)	0.838 (0.758‐0.895)
T‐TC	0.602 (0.583‐0.621)	0.454 (0.427‐0.481)	0.764 (0.740‐0.787)	0.678 (0.646‐0.708)	0.561 (0.537‐0.585)
T‐VC	0.598 (0.568‐0.627)	0.463 (0.422‐0.505)	0.755 (0.715‐0.792)	0.688 (0.639‐0.733)	0.547 (0.508‐0.584)

CytoNet, Cytology‐based multitask predictive Network; ACC, accuracy; SEN, sensitivity; SPE, specificity; PPV, positive predictive value; NPV, negative predictive value; TC, training cohort; IVC, internal validation cohort; EVC1, external validation cohort 1; EVC2, external validation cohort 2; C‐TC, cervical training cohort; C‐VC, cervical validation cohort; T‐TC, thyroid training cohort; T‐VC, thyroid validation cohort.

After LASSO regression analysis, clinical tumor stage, clinical lymph node stage, and radiological lymph node size were identified as significant variables associated with LNM. The clinical model incorporating these variables was constructed, and the integrated model was further developed by combining both the selected clinical predictors and the LNM‐predicted risk scores generated by CytoNet. As shown in Figure 2K, the integrated model demonstrated the highest discriminative performance in the combined cohort, achieving an AUC of 0.890 (95% CI 0.865‐0.915), which was significantly higher than that of the clinical model (AUC: 0.748, DeLong *p* < 0.001) and CytoNet alone (AUC: 0.858, DeLong *p* < 0.001, Table S4). The radar chart further indicated that both the integrated model and the CytoNet outperformed the clinical model and radiological assessment across the key performance metrics (Figure 2L, Table S5).

In the exploratory blinded reader study, CytoNet alone achieved an accuracy of 0.807, sensitivity of 0.757, and specificity of 0.823 for LNM prediction. When radiologists assessed LNM using routine clinical information and CT or MRI images alone, the pooled performance was lower, particularly among junior radiologists, with an accuracy of 0.616 and sensitivity of 0.441. Senior radiologists showed better unaided performance, with an accuracy of 0.716 and sensitivity of 0.577. After the assistance of CytoNet, the diagnostic performance of both junior and senior radiologists improved. Among junior radiologists, accuracy increased from 0.616 to 0.633, and sensitivity from 0.441 to 0.559. Among senior radiologists, accuracy increased from 0.716 to 0.731, and sensitivity from 0.577 to 0.649, with only minor changes in specificity (Table S6, Figure S5).

### 3.3 Performance of the Model for Predicting Prognosis in Bladder Cancer

The CytoNet demonstrated robust performance in predicting survival outcomes within the combined cohort, with cohort‐level C indices ranging from 0.680 to 0.710 for DFS and 0.644 to 0.706 for OS in the TC, IVC, EVC1, and EVC2 (Table 2). For DFS prediction, the time‐dependent C indices of CytoNet at 1 to 5 years remained stable across four cohorts (Figure 3A). Using the median predicted survival probability of 0.568 derived from the TC, patients were stratified into low‐risk and high‐risk groups. Kaplan–Meier analysis revealed significantly worse DFS in the high‐risk group across the TC, IVC, EVC1, and EVC2 (all log‐rank *p* < 0.01, Figure 3B). Similarly, for OS, the high‐risk group showed significantly lower survival probability compared to the low‐risk group across these four cohorts (all log‐rank *p* < 0.01, Figure 3C). The CytoNet also demonstrated superior performance for DFS prediction compared to the single‐task model, with C indices improving to a range of 0.680‐0.710 from 0.622–0.655 (Table S7).

**TABLE 2:** Performance of the CytoNet in predicting survival prognosis.

	TC	IVC	EVC1	EVC2	C‐TC	C‐VC
**DFS**						
C index (95% CI)	0.710 (0.669‐0.751)	0.693 (0.635‐0.750)	0.680 (0.619‐0.742)	0.689 (0.591‐0.786)	0.709 (0.653‐0.765)	0.690 (0.604‐0.776)
**OS**						
C index (95% CI)	0.706 (0.661‐0.750)	0.688 (0.621‐0.755)	0.644 (0.560‐0.728)	0.686 (0.582‐0.791)	0.670 (0.593‐0.747)	0.666 (0.541‐0.790)

CytoNet, Cytology‐based multitask predictive Network; TC, training cohort; IVC, internal validation cohort; EVC1, external validation cohort 1; EVC2, external validation cohort 2; C‐TC, cervical training cohort; C‐VC, cervical validation cohort; DFS, disease‐Free survival; OS, overall survival; C index, concordance index.

**FIGURE 3:**
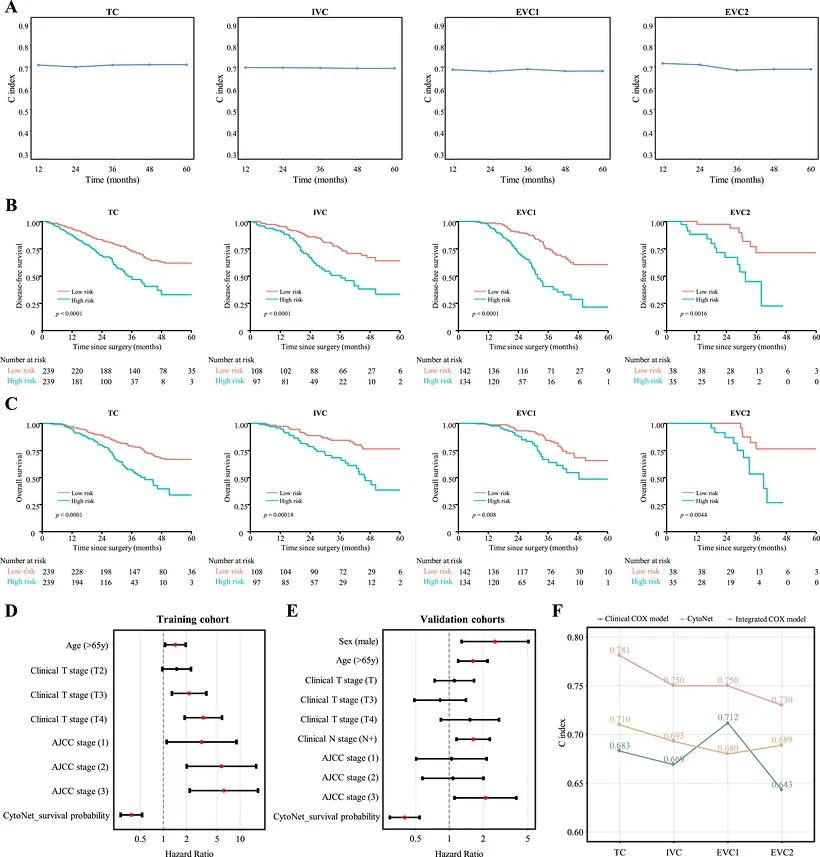
Model performance for predicting survival outcomes in bladder cancer. (A)​ Time‐dependent C indices of the CytoNet for disease‐free survival prediction at 1 to 5 years across different cohorts. (B,C)​ Kaplan‐Meier curves of disease‐free survival and overall survival for patients stratified by the CytoNet into low‐risk and high‐risk groups. *P*‐values were calculated using the log‐rank test. (D,E)​ Forest plots of multivariable Cox regression analyses for disease‐free survival in the training cohort and combined validation cohort, showing hazard ratios and 95% confidence intervals for clinical factors and survival probabilities generated by CytoNet. (F) Line charts compare the C indices for disease‐free survival prediction of the clinical Cox model, CytoNet, and the integrated Cox model across different cohorts. CytoNet, Cytology‐based multitask predictive Network; TC, training cohort; IVC, internal validation cohort; EVC1, external validation cohort 1; EVC2, external validation cohort 2; C index, concordance index; AJCC, American Joint Committee on Cancer.

To evaluate the independent prognostic value of the CytoNet, univariable and multivariable Cox regression analyses were conducted. The survival probabilities generated by the CytoNet were identified as a significant independent factor associated with DFS in both the TC (hazard ratio [HR] 0.38, 95% CI 0.28‐0.53, *p* < 0.001, Figure 3D) and the combined validation cohort (HR 0.40, 0.30‐0.54, *p* < 0.001, Figure 3E). The significant clinical factors (age, clinical T stage, AJCC stage) identified from the TC were used to construct a clinical Cox model. Subsequently, an integrated Cox model was developed by incorporating both these clinical factors and the CytoNet‐generated survival probabilities. Comparison of the models’ discriminative ability revealed that the integrated Cox model achieved superior C indices for DFS, followed by the CytoNet, while the clinical Cox model exhibited comparatively lower C indices (Figure 3F and Table S8).

Stratified analysis of AJCC stage III patients based on CytoNet risk classification suggested different patterns of adjuvant therapy benefit. For treatment‐benefit analysis, patients were grouped into class 1 (n = 100), combined classes 2 and 3 (n = 202), and class 4 (n = 112). Patients in class 1 showed no significant improvement in either DFS or OS with adjuvant therapy. In contrast, significant DFS and OS benefits were observed in combined classes 2 and 3 and in class 4 (log‐rank *p* < 0.05, Figure 4). However, in multivariable Cox interaction analyses adjusted for age, sex, pathological T stage, and pathological N stage, the interaction between adjuvant therapy and CytoNet‐defined risk group did not reach statistical significance for DFS or OS (*p* for interaction = 0.439 and 0.385, respectively). Therefore, this treatment‐benefit analysis should be interpreted as exploratory given the limited subgroup sample sizes and event numbers.

**FIGURE 4:**
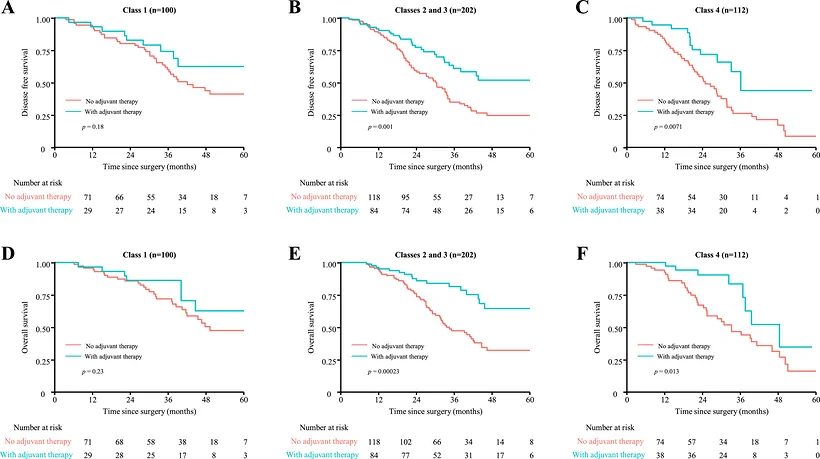
Assessment of adjuvant therapy benefit stratified by CytoNet in bladder cancer. Patients were stratified into four risk classes based on the CytoNet's dual‐task predictions: class 1 (low LNM risk and high survival score), class 2 (low LNM risk and low survival score), class 3 (high LNM risk and high survival score), and class 4 (high LNM risk and low survival score). (A‐C)​ Kaplan‐Meier curves of disease‐free survival for patients who are stratified according to receipt of adjuvant therapy in different risk classes. (D–F)​ Kaplan‐Meier curves of overall survival in different risk classes. CytoNet, Cytology‐based multitask predictive Network; LNM, lymph node metastasis.

### 3.4 Performance of the Model for Predicting LNM and Prognosis in IETC

To further assess the transportability of CytoNet beyond the Sun Yat‐sen University‐affiliated hospital system, the locked CytoNet was evaluated in the IETC. For LNM prediction, CytoNet maintained favorable discriminative performance, with an AUC of 0.833 (95% CI 0.742‐0.924), accuracy of 0.814, sensitivity of 0.783, specificity of 0.823, positive predictive value of 0.562, and negative predictive value of 0.929. Compared with radiological assessment, CytoNet showed better overall performance and reclassification improvement in the IETC. Calibration and decision curve analyses also demonstrated favorable agreement and clinical utility. For prognostic prediction, CytoNet achieved a C index of 0.704 (95% CI 0.633‐0.775) for DFS and 0.664 (0.566‐0.762) for OS. Risk stratification based on CytoNet further separated patients into groups with significantly different DFS and OS outcomes (log‐rank *p* = 0.0051 and *p* = 0.033, respectively; Table S9 and Figure S6).

### 3.5 Performance of the Model for Predicting LNM and Prognosis in Cervical and Thyroid Cancers

CytoNet demonstrated robust performance in the pan‐cytology evaluation for LNM prediction. For cervical cancer, the model achieved AUCs of 0.862 (95% CI 0.814‐0.910) in the C‐TC and 0.847 (0.764‐0.931) in the C‐VC. For thyroid cancer, the AUCs were 0.855 (95% CI 0.840‐0.870) in the T‐TC and 0.819 (95% CI, 0.793‐0.846) in the T‐VC (Figure 5A,B). The model‐derived risk scores effectively distinguished patients with and without LNM (Figure 5C,D). Calibration and decision curve analyses indicated well‐calibrated predictions and favorable clinical utility (Figure S7). The CytoNet showed a positive NRI and outperformed standard radiological assessment across multiple metrics (Figure 5E,F, Table 1, Table S10, Figure S8). Sankey diagrams illustrated good concordance between CytoNet predictions and pathological diagnoses (Figure 5G,H, Figure S9). Furthermore, in cervical cancer, the CytoNet's risk stratification revealed differential prognosis, with the high‐risk group showing a worse survival outcome (Figure 5I).

**FIGURE 5:**
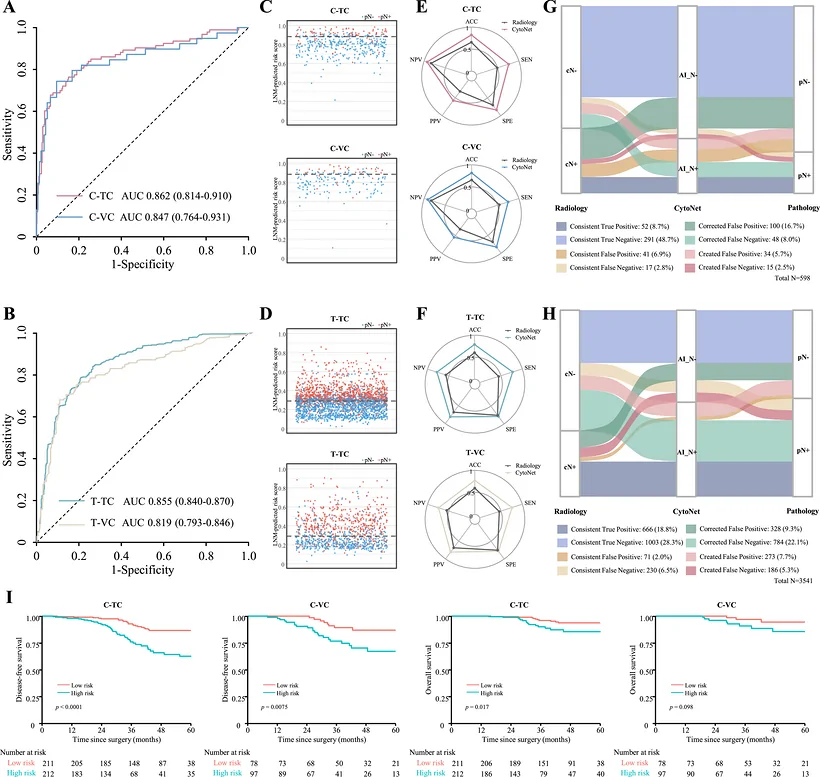
Pan‐cytology evaluation of CytoNet in cervical and thyroid cancers. (A,B) Receiver operating characteristic curves of CytoNet for predicting LNM across different cohorts. (C,D) Scatter plots of the LNM‐predicted risk scores. (E,F) Radar charts compare the performance for predicting LNM between radiology and CytoNet. (G,H) Sankey diagrams illustrating the concordance and reclassification relationships among radiological assessment, CytoNet prediction, and pathological lymph node status in the cervical cancer cohort and thyroid cancer cohort, respectively. The left, middle, and right columns represent radiology‐based nodal assessment, CytoNet‐predicted nodal status, and pathological reference status, respectively. cN‐/cN+ indicate radiology‐based negative or positive lymph node status; AI_N‐/AI_N+ indicate CytoNet‐predicted negative or positive lymph node status; and pN‐/pN+ indicate pathologically negative or positive lymph node status. The width of each flow is proportional to the number of patients. Flows are color‐coded to show consistent classifications, radiology misclassifications corrected by CytoNet, and new misclassifications introduced by CytoNet relative to radiology. (I) Kaplan‐Meier curves of disease‐free survival and overall survival for patients with cervical cancer stratified by the CytoNet into low‐risk and high‐risk groups. CytoNet, Cytology‐based multitask predictive Network; C‐TC, cervical training cohort; C‐VC, cervical validation cohort; T‐TC, thyroid training cohort; T‐VC, thyroid validation cohort; AUC, area under the curve; LNM, lymph node metastasis.

### 3.6 Interpretability Analysis of the Model

To elucidate the decision‐making process of CytoNet, a multifaceted interpretability analysis was conducted. UMAP visualization of the learned high‐dimensional cytopathological features revealed distinct clustering patterns between patients with and without LNM (Figure 6A). CAM heatmaps highlighted the specific cellular regions that drove the model's prediction, revealing distinct regions of model attention that correlated with nodal status (Figure 6B). In positive cases, the heatmaps predominantly focused on cell regions exhibiting invasive features, particularly those with elevated nuclear‐to‐cytoplasmic ratios, deep nuclear staining, coarse chromatin, and irregular nuclear membranes. Additionally, the model paid more attention to isolated or loosely arranged cells, which indicated the loss of cell adhesion and the aggressive growth pattern of metastatic potential. In contrast, the heatmaps of negative cases were more concentrated around cells with regular nuclear morphology and uniform chromatin distribution. These regions were characterized by tightly packed, papillary cell clusters with low nucleus‐to‐cytoplasm ratios, highlighting the absence of aggressive characteristics. Furthermore, SHAP analysis was employed to quantify feature importance. The bar plot and beeswarm plot demonstrated that the LNM risk score and survival probability generated by CytoNet were the most influential contributors to the model's output (Figure 6C,D).

**FIGURE 6:**
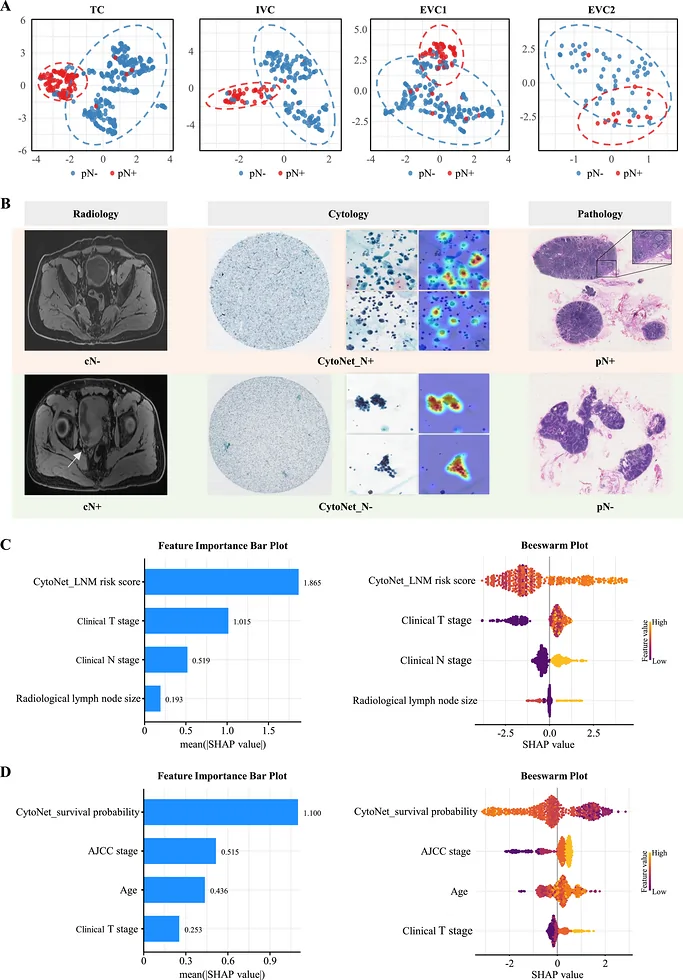
Model interpretability. (A) UMAP visualization of learned cytopathological features for LNM prediction. (B) Representative cases of diagnostic correction by CytoNet.​ CAM heatmaps highlight the key regions driving model decisions. (C,D) SHAP analysis to assess feature importance for model predictions. Bar plots and beeswarm plots visualize the contribution (SHAP value) of input variables to the model's output. UMAP, Uniform Manifold Approximation and Projection; LNM, lymph node metastasis; CytoNet, Cytology‐based multitask predictive Network; CAM, Class Activation Mapping; SHAP, SHapley Additive exPlanations.

## 4 Discussion

Accurately assessing LNM and survival prognosis is crucial for formulating individualized treatment strategies for patients with bladder, cervical, and thyroid cancers. This study developed and validated a multitask deep learning model named CytoNet based on cytopathological images, capable of predicting LNM risk and survival outcomes for these three cancer types within a unified framework. Through internal and external validation using multicenter cohorts, CytoNet demonstrated not only favorable predictive performance but also cross‐cancer generalization capability. To our knowledge, this is the first study to utilize cross‐cancer cytopathological images to evaluate the performance of a multitask deep learning framework in predicting LNM and prognosis.

Predicting LNM and survival outcomes in cancer patients faces multiple challenges, including insufficient accuracy, high tumor heterogeneity, and complex prognostic factors. Given the significant biological and morphological differences among different cancer types, developing a universal model with broad generalizability is particularly difficult. Previous studies have predominantly focused on single cancer types or single clinical tasks, restricting their clinical application potential [19, 20, 21, 36]. This study adopted a multitask architecture, enabling CytoNet to learn cytopathological representations relevant to both LNM and survival prognosis in a coordinated manner. The results confirmed that the model exhibited robust predictive performance across all cancer cohorts, with AUC values for LNM prediction exceeding 0.8 and C indices for survival prediction also showing good discriminatory ability, outperforming the corresponding single‐task models. The cross‐cancer applicability of CytoNet may be attributed to shared cytomorphological hallmarks of tumor aggressiveness. Although bladder, cervical, and thyroid cancers differ in tissue origin and biological behavior, aggressive tumors across these cancer types may show convergent cytological patterns, including increased nuclear‐to‐cytoplasmic ratios, nuclear hyperchromasia, coarse chromatin, irregular nuclear membranes, cellular pleomorphism, and reduced intercellular cohesion. These features may reflect common malignant processes related to proliferation, genomic instability, and metastatic potential. Consistent with this hypothesis, CAM analysis showed that, in LNM‐positive cases, CytoNet preferentially focused on cytological regions with invasive features, including high nuclear‐to‐cytoplasmic ratios, nuclear hyperchromasia, coarse chromatin, irregular nuclear membranes, and isolated or loosely arranged cells. Thus, the pan‐cytology validation suggests that CytoNet may capture shared aggressiveness‐related morphological signals while preserving cancer‐specific adaptation.

Preoperative LNM status is a critical determinant for patient treatment decisions. For bladder cancer, surgical options include​ standard and extended lymph node dissection, though​ the optimal extent of dissection is clinically debated. Extended dissection provides more accurate nodal staging but increases the risk of postoperative complications (e.g., lymphocele and nerve injury). Although some studies suggest extended dissection may improve cancer‐specific survival and recurrence‐free survival, its benefit for DFS or OS remains unconfirmed [2, 37, 38]. Therefore, in current clinical practice, extended dissection is used for patients preoperatively assessed as high risk for LNM, while standard dissection is used for low‐risk patients. However, accurately assessing LNM preoperatively remains challenging. The accuracy of traditional imaging methods (CT or MRI) needs improvement. Imaging relies on macroscopic morphological changes in lymph nodes, and its sensitivity and specificity are often limited by resolution and interference from benign conditions, potentially leading to undertreatment or overtreatment. Machine learning or deep learning models based on clinical factors and imaging also have limited predictive efficacy [36, 39, 40, 41]. In this study, the CytoNet analyzed cytopathological images to capture microscopic morphological abnormalities in cancer cells, which may precede macroscopic changes in imaging. In validation cohorts and test cohort, CytoNet's comprehensive performance surpassed conventional imaging assessments, highlighting its potential to overcome the limitations of current preoperative staging methods and providing a more precise tool for early LNM risk assessment. Importantly, CytoNet is not intended to replace postoperative histopathological assessment, which remains the definitive reference standard for nodal staging. Rather, its clinical value lies in providing preoperative cytology‐derived metastatic risk information at a time when pathological nodal status is not yet available. In this setting, CytoNet may complement CT/MRI‐based assessment, improve the recognition of patients at high risk for pathological LNM, and support individualized decisions regarding lymph node dissection, perioperative treatment, and follow‐up intensity. This complementary value was further supported by the exploratory blinded reader study, in which CytoNet assistance improved radiologists’ accuracy and sensitivity for preoperative LNM assessment.

The feasibility of multimodal integration was supported by the integrated model. Although the present study was designed to establish a cytology image‐based predictive framework, combining CytoNet‐derived LNM risk scores with selected clinical and radiological variables further improved discriminative performance compared with either the clinical model or CytoNet alone. This finding suggests that cytology‐derived microscopic morphological information is complementary to routinely available clinical and imaging features. Future studies may extend this strategy by developing end‐to‐end multimodal frameworks that integrate cytology WSIs with CT or MRI images, and, when clinically available, histopathological or molecular data, to further improve predictive performance and biological interpretability.

Furthermore, the LNM status is an important factor in determining bladder‐preserving treatment strategies. For patients undergoing neoadjuvant therapy, the presence of LNM after treatment typically indicates a higher risk of local recurrence and distant metastasis. In such cases, opting for bladder preservation may not achieve optimal tumor control, and radical cystectomy with lymph node dissection may represent a more prudent therapeutic choice [42]. In the neoadjuvant therapy subgroup, the accuracy of CytoNet for LNM prediction was 80.8%, surpassing the traditional radiological assessment (60.6%). This enhanced diagnostic capability provides valuable decision support for selecting post‐neoadjuvant treatment strategies.

To further contextualize these findings, we compared CytoNet with representative AI‐based models previously developed for bladder cancer LNM prediction (Table S11). Although direct comparison across studies should be interpreted cautiously due to differences in patient cohorts, input modalities, and validation strategies, most prior studies focused on single‐task prediction using CT, MRI, histopathological WSIs, or clinical variables. In contrast, CytoNet leverages preoperative cytology WSIs for multitask prediction of both LNM and DFS, supports AI‐assisted reader evaluation, and demonstrates cross‐cancer validation across bladder, cervical, and thyroid cancer cohorts. These features highlight the complementary positioning of CytoNet relative to previously reported models.

Although the C index of CytoNet for DFS prediction was moderate, no universally accepted minimum C index threshold has been established to define clinical usefulness for bladder cancer prognostic models. Its clinical relevance should therefore be interpreted according to its intended use and incremental value. In this study, CytoNet provided prognostic information from preoperative cytology images alone, remained independently associated with DFS, improved the integrated Cox model when combined with clinical factors, and consistently stratified patients into distinct survival risk groups. Thus, CytoNet may serve as a complementary risk stratification and decision‐support tool rather than a standalone prognostic determinant. The clinical application potential of CytoNet is further reflected in its potentially actionable risk stratification. For bladder cancer patients with AJCC Stage III disease, the benefit‐risk profile of postoperative adjuvant therapy remains controversial [3]. Several studies indicate potential survival benefits, but it may also lead to overtreatment and associated side effects in some patients [43, 44, 45, 46]. By integrating CytoNet's LNM risk score and survival probability, we stratified patients into four distinct risk classes. Kaplan‐Meier analyses suggested that patients in the intermediate‐risk and high‐risk groups (classes 2, 3, and 4) showed apparent survival benefits from adjuvant therapy, while no significant benefit was observed in the low‐risk group (class 1). This suggests that CytoNet has the potential to serve as a clinical decision‐support tool to help​ guide adjuvant therapy evaluation. Whereas​ intensified treatment and close follow‐up could be considered for patients in classes 2‐4, treatment de‐escalation might be justified for those in class 1 to spare them unnecessary toxicity. This CytoNet‐based risk stratification strategy could help shift the current adjuvant therapy paradigm toward a more precise selection of patients who would benefit, thereby facilitating individualized treatment.

We conducted a multi‐level interpretability analysis to investigate the rationality of CytoNet's decisions. UMAP visualization demonstrated that the high‐dimensional features extracted by CytoNet could clearly separate LNM‐positive and LNM‐negative patients, indicating its ability to identify​ pathological features related to nodal status. CAM heatmaps further localized the model's attention to pathologically significant regions, such as those with high nucleus‐to‐cytoplasm ratios and specific cellular arrangements, thereby enhancing​ decision transparency and clinical interpretability. SHAP analysis quantified feature contributions, revealing that the LNM risk score and survival probability derived from CytoNet were key factors​ for predicting LNM and prognosis, surpassing traditional clinical staging. This confirms that the model can identify novel features independent of known factors.

This study has several limitations. First, the retrospective design may introduce selection bias. Across the bladder, cervical, and thyroid cancer cohorts, eligible patients were required to have available cytology slides, imaging data, pathological lymph node status, and clinical outcome information, which may limit the representativeness of the study population. In particular, the bladder cancer cohort consisted of patients who underwent radical cystectomy with lymph node dissection and had complete preoperative cytology and imaging data; therefore, it may not fully represent the broader bladder cancer population, such as patients treated with bladder‐preserving strategies, those without cytology specimens, or those with incomplete imaging or follow‐up information. Although multicenter validation was performed to improve generalizability, prospective studies with consecutive patient enrollment are still required to further validate the clinical utility of CytoNet. In addition, some external validation datasets remained relatively small, including EVC2 (n = 73) and the independent test cohort from non‐Sun Yat‐sen University‐affiliated hospitals (n = 102), which may reduce statistical power and lead to wider confidence intervals or less stable estimates of event‐dependent metrics. Therefore, these results should be interpreted as supportive external validation, and larger validation across geographically diverse institutions is still warranted. Second, although generalizability was validated across three cancer types, the model's effectiveness in other cancers requires exploration. Third, although the integrated model suggested the potential value of combining cytology‐derived features with clinical and radiological variables, end‐to‐end multimodal fusion of cytology WSIs, CT or MRI images, histopathology, and molecular data still requires further investigation. Fourth, race and ethnicity were not available because these variables were not routinely recorded in the institutional medical records, which prevented us from assessing their potential confounding effects. Future validation in more ethnically diverse populations is warranted. Finally, the biological links between the morphological features identified by the model and specific molecular pathways remain unclear and warrant further investigation through integration with multi‐omics data.

In conclusion​, CytoNet, a cytology image‐based multitask predictive network, provides accuracy and generalizable prediction of LNM and survival outcomes across bladder, cervical, and thyroid cancers. The model's clinical risk stratification combining with interpretability analysis, offers a useful tool for clinical practice and individualized cancer management.

## Author Contributions

SW, TL, GH, FJ, and YX conceived and designed the study. RS, YW, MZ, HW, HY, YN, and NO contributed to data acquisition. XH, ZL, and RC performed artificial intelligence analysis. GH, FJ, and YX conducted data analysis and interpretation. GH and SW drafted and revised the manuscript. SW, TL, GH, FJ, and YX accessed and verified the data reported in the manuscript. SW and TL provided study supervision. All authors had full access to all the data reported in the study and had final responsibility for the decision to submit for publication.

## Conflicts of Interest

The authors declare no conflicts of interest.

## Supporting information




**Supporting File**: advs76577‐sup‐0001‐SuppMat.pdf.

## Data Availability

To protect patient privacy, the cytology image dataset and other patient‐related information from the in‐house collection are not publicly accessible. However, these data can be provided upon reasonable request by contacting the corresponding author via email. A signed data access agreement is required before data sharing. The project code is available at https://github.com/cellsvision/CytoNet.
