# Supplementary material for: Multitask Deep Learning Model for Predicting Lymph Node Metastasis and Disease‐Free Survival From Cytology Images in Bladder Cancer: A Multicenter Study With Pan‐Cytology Validation
Source: Adv Sci (Weinh). 2026 Oct 21:e76577. Online ahead of print. doi: 10.1002/advs.76577 (PMC13685576; doi:10.1002/advs.76577)
Supplement: Supplementary file 1 [file ADVS-9999-e76577-s001.pdf]

Supplementary Material

Supplementary Methods 3

Inclusion and exclusion criteria for patients with bladder cancer 3

Sample size calculation 3

Proposed multitask deep learning network architecture 4

Supplementary Tables 10

Supplementary Table 1. Characteristics of patients with bladder cancer in the training and validation cohorts 10

Supplementary Table 2. Characteristics of patients with cervical cancer and thyroid cancer in the training and validation cohorts 12

Supplementary Table 3. Calibration analysis of CytoNet in predicting lymph node metastasis 13

Supplementary Table 4. DeLong test for pairwise AUC comparisons among the clinical model, CytoNet, and the integrated model in the combined cohort 13

Supplementary Table 5. Assessment of the predictive performance for lymph node metastasis in the combined cohort 13

Supplementary Table 6. Diagnostic performance of CytoNet, radiologists alone, and CytoNet-assisted radiologists in predicting lymph node metastasis 14

Supplementary Table 7. Comparison of CytoNet and the single-task model for disease-free survival prediction in bladder cancer 15

Supplementary Table 8. Assessment of model performance for disease-free survival prediction in bladder cancer 15

Supplementary Table 9. Performance of the CytoNet and radiology in predicting lymph node metastasis in the independent external test cohort 15

Supplementary Table 10. Performance of radiology in predicting lymph node metastasis 16

Supplementary Table 11. Comparison of CytoNet with representative AI-based models for lymph node metastasis prediction in bladder cancer........17
Supplementary Figures 19
Supplementary Figure 1. Flowchart of patient selection and study cohort allocation..19
Supplementary Figure 2. Sankey diagrams visualize the concordance among radiology assessment, CytoNet prediction, and pathological diagnosis in different bladder cancer cohorts..20
Supplementary Figure 3. Performance of the CytoNet for lymph node metastasis prediction in the combined cohort 21
Supplementary Figure 4. Performance of CytoNet versus radiology for lymph node metastasis detection after neoadjuvant therapy 22
Supplementary Figure 5. Performance changes of radiologists with CytoNet assistance for preoperative lymph node metastasis prediction......................23
Supplementary Figure 6. Independent external testing of CytoNet in non-Sun Yat-sen University-affiliated hospitals..25
Supplementary Figure 7. Model performance for predicting lymph node metastasis in cervical and thyroid cancers 26
Supplementary Figure 8. Comprehensive performance comparison between radiology and the CytoNet for lymph node metastasis prediction in cervical and thyroid cancers...27
Supplementary Figure 9. Sankey diagrams visualize the concordance among radiology assessment, CytoNet prediction, and pathological diagnosis across cervical and thyroid cancer cohorts 28

Supplementary Methods

Inclusion and exclusion criteria for patients with bladder cancer

The inclusion criteria were as follows: (a) histologically confirmed bladder cancer and underwent radical cystectomy with pelvic lymph node dissection; (b) availability of urine liquid-based cytology slides obtained within 3 months before surgery; and (c) availability of preoperative pelvic computed tomography (CT) or magnetic resonance imaging (MRI) examinations.

The exclusion criteria were: (a) absence of a urine cytology examination within 3 months prior to surgery at the participating medical centers; (b) ineligible image quality of cytology slides (e.g., insufficient number of tumor cells, severe fading or presence of artifacts) that precluded image analysis; (c) unavailability of preoperative pelvic CT or MRI examinations; or (d) incomplete baseline clinical data or follow-up information.

Sample size calculation

The sample size was calculated using PASS 15 software. For the task of predicting lymph node metastasis (LNM), the sample size calculation was performed for the comparison of the area under the receiver operating characteristic curve (AUC). The parameters were set as follows: a two-sided significance level (α) of 0.05, a statistical power ($1-\beta$) of 0.90, a null hypothesis AUC (AUC0) of 0.70, and an alternative hypothesis AUC (AUC1) of 0.80. Based on the reported incidence of LNM in our cohort, which is approximately 20%, the ratio of negative to positive cases (R=N-/N+) was set to 4. The results indicated that a minimum of 95 positive cases (LNM present) and 380 negative cases (LNM absent), yielding a total sample size of 475 cases, was required to achieve the desired power. For the task of predicting disease-free survival (DFS), a Cox proportional hazards regression model was applied for the sample size calculation. The parameters were set as follows: a two-sided significance level (α) of 0.05, a statistical power ($1-\beta$) of 0.90, and an overall event rate (P) of 0.35, which was based on the observed DFS rate in our cohort. The calculation assumed a hazard ratio (HR) of 0.40 for the predictor variable, corresponding to a log hazard ratio (B) of ln (0.40) = -0.9163. The standard deviation (S) of the predictor was 0.256, and the R-

squared was 0. The analysis indicated that a minimum of 546 cases was required to detect the specified effect with sufficient power. In this study, a total of 1134 patients were ultimately enrolled, which exceeds the minimum requirement and ensures adequate power for the analysis.

Proposed multitask deep learning network architecture

1. Image preprocessing

The analysis of high-resolution whole-slide images (WSIs, typically up to 50000 × 50000 pixels) employs a multi-stage pipeline. The initial preprocessing stage involves splitting each WSI into smaller, manageable patches using a sliding window algorithm without overlap.

For self-supervised pretraining: WSIs are segmented into non-overlapping patches of 512 × 512 pixels. This size is computationally efficient for processing the large scale of unlabeled data required for this stage and is compatible with the input size of the RegNetY backbone network.

For supervised fine-tuning: To leverage expert annotations, a distinct patching strategy is used. For each WSI containing annotations of different types of cells, 1024 × 1024 pixel patches are extracted, centered on the provided annotation coordinates. This larger patch size provides richer contextual information around the annotated cells, which is crucial for effective model fine-tuning. Both patch sizes ensure compatibility with the subsequent model architectures.

2. Self-supervised pretraining

2.1 Backbone Network Architecture

We developed a general feature extraction model for cytopathological images, with RegNetY as the core architecture. The feature extractor is defined as a function:

$$f_\theta: \mathbb{R}^{s\times s\times 3} \rightarrow \mathbb{R}^d$$

where θ represents the model parameters, an input image is of spatial size $s \times s$ with 3 color channels, and d is the dimensionality of the output feature vector. RegNetY

was selected for its optimized balance between computational efficiency and feature representation capacity, making it suitable for complex medical images.

2.2 DINO framework

The DINO (self-**DI**stillation with **NO** labels) framework was employed for self-supervised pretraining. This approach combines knowledge distillation and contrastive learning, enabling the model to learn general visual representations from unlabeled data. Specifically, for an input image x, multiple augmented views were generated: two global views (x_g^1, x_g^2) and n local views ($x_l^1, \ldots, x_l^n$). These views are processed through twin networks: a student network f_s and a teacher network f_t.

The teacher network's parameters (θ_t) are updated from the student's parameters (θ_s) via an exponential moving average (EMA), governed by a momentum coefficient m:

$$\theta_t \leftarrow m \cdot \theta_t + (1 - m) \cdot \theta_s$$

The momentum m is increased during training from an initial value of 0.996 to nearly 1, ensuring stable evolution of the teacher network.

2.3 Loss Function

The core training objective is to maximize the similarity between the outputs of the teacher and student networks for different augmented views of the same image. The outputs are K-dimensional feature vectors, normalized using a softmax function parameterized by a temperature constant τ. The similarity is measured using the loss function:

$$\mathcal{L}_{CE} = -\sum_{k=1}^{K} p_t^{(k)} \log (p_s^{(k)})$$

where $p_t^{(k)}$ is the output probability distribution of the teacher network (serving as the target), and $p_s^{(k)}$ is the output of the student network for the k-th dimension. A stop-gradient (sg) operation is applied to the teacher's output, meaning gradients are calculated and used to update only the student network's parameters via backpropagation. The teacher network is updated solely via the EMA of the student's parameters.

2.4 Data Augmentation Strategy

A diverse set of augmentations was applied to facilitate robust feature learning:

Weak augmentations: Random horizontal/vertical flipping (probability = 0.5).

Strong augmentations: Color jittering (brightness ±0.4, contrast ±0.4, saturation ±0.2, hue ±0.1; probability=0.8), random grayscale (*p*=0.2), Gaussian blur (*p*=1.0 for global/0.1 for local views), and Solarization (*p*=0.2).

Size cropping: Global views were scaled between 0.4 and 1.0 of the original size, while local views were scaled between 0.05 and 0.4.

The pr-training dataset consisted of over 100000 unlabeled cytology images from Sun Yat-sen Memorial Hospital of Sun Yat-sen University, encompassing various tissue origins (urine, cervical specimens, and thyroid fine-needle aspirations).

3. Supervised fine-tuning

3.1 Network Architecture Enhancement

Building upon the DINO-pretrained model, we integrated a Convolutional Block Attention Module (CBAM) into the RegNetY architecture to enhance its focus on relevant pathological features. The CBAM module applies channel and spatial attention mechanisms, enabling adaptive feature recalibration. The fine-tuning network is defined as:

$$f_{\text{fine-tune}}(x) = \text{CBAM}(\text{RegNetY}(x))$$

3.2 Loss function and optimization

The fine-tuning stage utilizes a standard cross-entropy loss:

$$\mathcal{L}_{CE} = -\sum_{c=1}^{C} y_c \log(p_c)$$

Here, y_c is the true label (C=6, including high-grade urothelial carcinoma, atypical urothelial cells, normal urothelial cells, histiocytes, impurities, and blank regions), and p_c is the predicted probability.

The model was optimized using the Adam optimizer with an initial learning rate ($lr = 5 \times 10^{-6}$). A CosineAnnealingWarmRestarts scheduler was used to adjust the learning rate:

$$lr(t) = \eta_{\min} + \frac{1}{2}(\eta_{\max} - \eta_{\min})(1 + \cos(\frac{T_{cur}}{T_i}\pi))$$

where T_{cur} is the current iteration count, T_i is the period length, and $\eta_{\max} = 10^{-5}$, $\eta_{\min} = 10^{-6}$. The scheduler was restarted every 3 epochs.

4. Multitask model for lymph node metastasis prediction and survival analysis

4.1 Top-N Feature (TNF) Model Architecture

The TNF model employs a two-level multiple instance learning (MIL) architecture, treating each WSI as a "bag" containing numerous feature "instances" (i.e., patches). Given input features $X \in \mathbb{R}^{N \times d}$ (where N is the number of patches and d is the feature dimension), the TNF model first groups patches by their predicted cell category $c \in \{1,2,\ldots,C_{box}\}$. For each category c, the top K (K=200) instances with the highest prediction confidence are selected to form a sub-bag, $X_c = \{x_i^c\}_{i=1}^{K}$.

An aggregation function with a learnable attention mechanism then computes a composite feature vector for each category:

$$z_c = \sum_{i=1}^{K} \alpha_i^c f_{\text{dense}}(x_i^c)$$

The attention weight α_i^c for each instance is calculated as:

$$\alpha_i^c = \frac{\exp\left(a(x_i^c)\right)}{\sum_j \exp\left(a(x_j^c)\right)}$$

$$a(x) = w^T \tanh(Vx) \odot \sigma(Ux) + b$$

Here, w, V, U are learnable parameters, and $\odot$ denotes element-wise multiplication. The final WSI-level representation is obtained by concatenating the aggregated features from all cell categories:

$$z = \text{concat}([z_1, z_2, \ldots, z_{C_{box}}])$$

4.2 Multitask learning branches

Two prediction branches share the unified WSI representation.

4.2.1 Lymph node metastasis classification

This branch predicts the probability of lymph node metastasis using a softmax classifier:

$$P_{\text{cls}} = \text{softmax}(W_c z + b_c)$$

It was trained with a cross-entropy loss:

$$\mathcal{L}_{\text{cls}} = -\sum_{j=1}^{C} y_j \log\left(P_{\text{cls},j}\right)$$

4.2.2 Survival Analysis

This branch predicts a patient-specific risk value:

$$r = W_s z + b_s$$

It was trained using the negative log partial likelihood loss of the Cox proportional hazards model:

$$\mathcal{L}_{\text{cox}} = -\frac{1}{N_{\text{events}}} \sum_{i:y_i=1} \left(r_i - \log\left(\sum_{j\in\mathcal{R}(t_i)} e^{r_j}\right)\right)$$

where $\mathcal{R}(t_i)$ is the set of patients still at risk at time t_i, and y_i indicates the event status.

For subsequent analysis, the risk value r is mapped to a survival propensity probability using the transformation:

$$P = \frac{1}{1 + e^{r}}$$

The total multitask loss is a weighted sum:

$$\mathcal{L}_{\text{total}} = \alpha\mathcal{L}_{\text{cls}} + \beta\mathcal{L}_{\text{cox}}$$

where the balancing coefficients α and β were both set to 1.0.

4.3 Training strategy

The multitask model was trained using the RAdam optimizer with an initial learning rate of 5×10^{-6} and the same CosineAnnealingWarmRestarts scheduler as described in 3.2. To mitigate class imbalance in the metastasis classification task, oversampling of positive samples was performed.

5. Interpretability analysis

Class Activation Mapping (CAM) was used to visualize the pathological regions most influential for the model's predictions. The attention weights (w_i) and prediction confidences (c_i) from the TNF model are projected back onto the original WSI coordinates. The resulting saliency map $M(p)$ for a point p on the WSI is computed as:

$$M(p) = \sum_{i \in \mathcal{N}(p)} w_i \cdot c_i$$

where $\mathcal{N}(p)$ is the set of patches neighboring location p.

6. Pan-cytology validation methodology

To evaluate the generalizability and robustness of the proposed framework beyond bladder cancer, a pan-cytology validation was conducted on two additional cancer types: cervical cancer and thyroid cancer. The validation strategy adhered to the following principled framework:

6.1 Shared self-supervised pretraining:

The same foundation model, pretrained in a self-supervised manner (via DINO) on a large, diverse cohort of pan-cytology images (including urine, cervix, and thyroid origins), was utilized as the universal feature extractor.

6.2 Cytology type-specific supervised fine-tuning

For each target cancer type (cervical and thyroid), the shared pretrained model was subsequently fine-tuned separately. This critical step involved each cancer's own set of meticulously annotated cell-level data. The fine-tuning process adapts the general feature extractor to the specific cytopathological characteristics (e.g., nuclear features, cytoplasmic patterns) unique to cervical and thyroid cancers, thereby tailoring the model for the target domain while leveraging shared foundational knowledge.

6.3 Multitask prediction:

For each fine-tuned cytology-specific feature extraction model, the image features were extracted from the respective WSIs. These features were then fed into the TNF multitask model for independent training and validation. The TNF model was trained and evaluated to predict lymph node metastasis status or disease-free survival.

Supplementary Tables

Supplementary Table 1. Characteristics of patients with bladder cancer in the training and validation cohorts

	TC (n=478)	IVC (n=205)	EVC1 (n=276)	EVC2 (n=73)	IETC (n=102)
Age (years)	65 (57-73)	63 (57-71)	68 (62-73)	68 (61-75)	67 (61-75)
Sex					
Female	67 (14.0%)	26 (12.7%)	39 (14.1%)	9 (12.3%)	18 (17.6%)
Male	411 (86.0%)	179 (87.3%)	237 (85.9%)	64 (87.7%)	84 (82.4%)
Radiological tumor size (cm)					
<1.5	136 (28.5%)	70 (34.1%)	87 (31.5%)	10 (13.7%)	35 (34.3%)
1.5-3	125 (26.2%)	32 (15.6%)	81 (29.3%)	25 (34.2%)	25 (24.5%)
>3	217 (45.4%)	103 (50.2%)	108 (39.1%)	38 (52.1%)	42 (41.2%)
Radiological tumor number					
Single	228 (47.7%)	119 (58.0%)	132 (47.8%)	31 (42.5%)	53 (52.0%)
Multiple	250 (52.3%)	86 (42.0%)	144 (52.2%)	42 (57.5%)	49 (48.0%)
Radiological lymph node size (mm)					
<5	413 (86.4%)	170 (82.9%)	207 (75.0%)	60 (82.2%)	78 (76.5%)
5-10	36 (7.5%)	29 (14.1%)	51 (18.5%)	10 (13.7%)	20 (19.6%)
>10	29 (6.1%)	6 (2.9%)	18 (6.5%)	3 (4.1%)	4 (4.0%)
Clinical T stage					
cTa-T1	166 (34.7%)	70 (34.1%)	90 (32.6%)	28 (38.4%)	32 (31.4%)
cT2	195 (40.8%)	88 (42.9%)	111 (40.2%)	15 (20.5%)	43 (42.2%)
cT3	77 (16.1%)	30 (14.6%)	51 (18.5%)	26 (35.6%)	17 (16.7%)
cT4	40 (8.4%)	17 (8.3%)	24 (8.7%)	4 (5.5%)	10 (9.8%)
Clinical N stage					
cN0	359 (75.1%)	151 (73.7%)	161 (58.3%)	45 (61.6%)	64 (62.7%)
cN1-3	119 (24.9%)	54 (26.3%)	115 (41.7%)	28 (38.4%)	38 (37.3%)
Pathological T stage					
pTa-T1	199 (41.6%)	80 (39.0%)	69 (25.0%)	14 (19.2%)	32 (31.4%)
pT2	103 (21.5%)	58 (28.3%)	129 (46.7%)	42 (57.5%)	37 (36.3%)
pT3	112 (23.4%)	47 (22.9%)	63 (22.8%)	15 (20.5%)	25 (24.5%)
pT4	64 (13.4%)	20 (9.8%)	15 (5.4%)	2 (2.7%)	8 (7.8%)
Pathological N stage					
pN0	374 (78.2%)	160 (78.0%)	219 (79.3%)	59 (80.8%)	79 (77.5%)
pN1-3	104 (21.8%)	45 (22.0%)	57 (20.7%)	14 (19.2%)	23 (22.5%)
AJCC stage					
0	55 (11.5%)	20 (9.8%)	42 (15.2%)	8 (11.0%)	15 (14.7%)
I	138 (28.9%)	54 (26.3%)	15 (5.4%)	5 (6.8%)	14 (13.7%)
II	90 (18.8%)	48 (23.4%)	108 (39.1%)	35 (47.9%)	28 (27.5%)
III	195 (40.8%)	83 (40.5%)	111 (40.2%)	25 (34.2%)	45 (44.1%)
Postoperative adjuvant therapy					

No	399 (83.5%)	162 (79.0%)	225 (81.5%)	65 (89.0%)	84 (82.4%)
Yes	79 (16.5%)	43 (21.0%)	51 (18.5%)	8 (11.0%)	18 (17.6%)
DFS (months)					
Follow-up time	29 (17-42)	31 (20-45)	29 (20-39)	28 (16-35)	20 (15-29)
OS (months)					
Follow-up time	30 (19-43)	35 (22-46)	31 (20-41)	29 (19-36)	22 (17-30)

Data are median (IQR) or n (%). TC, training cohort; IVC, internal validation cohort; EVC1, external validation cohort 1; EVC2, external validation cohort 2; IETC, independent external test cohort; AJCC, American Joint Committee on Cancer; DFS, disease-Free survival; OS, overall survival.

Supplementary Table 2. Characteristics of patients with cervical cancer and thyroid cancer in the training and validation cohorts

	Cervical cancer (n=598)		Thyroid cancer (n=3541)	
	Training cohort (n=423)	Validation cohort (n=175)	Training cohort (n=2514)	Validation cohort (n=1027)
Age (years)	51 (45-58)	53 (43-58)	42 (33-52)	42 (34-52)
Sex				
Female	423 (100%)	175 (100%)	1915 (76.2%)	778 (75.8%)
Male	NA	NA	599 (23.8%)	249 (24.2%)
Clinical N stage				
cN-	279 (66.0%)	111 (63.4%)	1635 (65.0%)	655 (63.8%)
cN+	144 (34.0%)	64 (36.6%)	879 (35.0%)	372 (36.2%)
Pathological N stage				
pN-	330 (78.0%)	136 (77.7%)	1201 (47.8%)	474 (46.2%)
pN+	93 (22.0%)	39 (22.3%)	1313 (52.2%)	553 (53.8%)
DFS (months)				
Follow-up time	37 (24-52)	37 (25-55)	NA	NA
OS (months)				
Follow-up time	38 (27-55)	38 (26-55)	NA	NA

Data are median (IQR) or n (%). NA, not applicable; DFS, disease-Free survival; OS, overall survival.

Supplementary Table 3. Calibration analysis of CytoNet in predicting lymph node metastasis

Cohorts	Brier score	95% CI
Training cohort	0.106	0.089-0.121
Internal validation cohort	0.108	0.086-0.135
External validation cohort 1	0.119	0.097-0.142
External validation cohort 2	0.113	0.074-0.159
Cervical training cohort	0.108	0.089-0.126
Cervical validation cohort	0.116	0.091-0.145
Thyroid training cohort	0.156	0.148-0.164
Thyroid validation cohort	0.169	0.157-0.182

CytoNet, Cytology-based multitask predictive Network.

Supplementary Table 4. DeLong test for pairwise AUC comparisons among the clinical model, CytoNet, and the integrated model in the combined cohort

Comparison	AUC 1	AUC 2	*p* value
Clinical model vs CytoNet	0.748	0.858	$p<0.001$
Clinical model vs Integrated model	0.748	0.890	$p<0.001$
CytoNet vs Integrated model	0.858	0.890	$p<0.001$

CytoNet, Cytology-based multitask predictive Network; AUC, area under the curve.

Supplementary Table 5. Assessment of the predictive performance for lymph node metastasis in the combined cohort

Methods	AUC (95% CI)	Accuracy (95% CI)	Sensitivity (95% CI)	Specificity (95% CI)	Positive predictive value (95% CI)	Negative predictive value (95% CI)
Radiology	-	0.698 (0.669-0.725)	0.509 (0.443-0.574)	0.749 (0.718-0.777)	0.354 (0.304-0.409)	0.849 (0.821-0.874)
Clinical	0.748 (0.712-0.784)	0.735 (0.708-0.761)	0.577 (0.511-0.641)	0.778 (0.749-0.805)	0.414 (0.360-0.470)	0.872 (0.845-0.894)
CytoNet	0.858 (0.828-0.888)	0.833 (0.809-0.855)	0.791 (0.732-0.839)	0.845 (0.818-0.868)	0.580 (0.523-0.634)	0.937 (0.917-0.953)
Integrated	0.890 (0.865-0.915)	0.823 (0.798-0.845)	0.832 (0.777-0.875)	0.820 (0.792-0.845)	0.556 (0.502-0.609)	0.947 (0.928-0.962)

CytoNet, Cytology-based multitask predictive Network; AUC, area under the curve.

Supplementary Table 6. Diagnostic performance of CytoNet, radiologists alone, and CytoNet-assisted radiologists in predicting lymph node metastasis

	Accuracy (95% CI)	Sensitivity (95% CI)	Specificity (95% CI)	Positive predictive value (95% CI)	Negative predictive value (95% CI)
CytoNet	0.807 (0.736-0.862)	0.757 (0.599-0.866)	0.823 (0.742-0.882)	0.583 (0.443-0.712)	0.912 (0.841-0.953)
Radiologists alone					
Junior radiologist 1	0.633 (0.544-0.706)	0.432 (0.287-0.591)	0.699 (0.609-0.776)	0.320 (0.208-0.458)	0.790 (0.700-0.858)
Junior radiologist 2	0.613 (0.533-0.688)	0.514 (0.359-0.665)	0.646 (0.554-0.728)	0.322 (0.217-0.449)	0.802 (0.709-0.871)
Junior radiologist 3	0.600 (0.520-0.675)	0.378 (0.241-0.539)	0.673 (0.582-0.752)	0.275 (0.171-0.410)	0.768 (0.675-0.840)
All junior radiologists	0.616 (0.570-0.659)	0.441 (0.353-0.534)	0.673 (0.621-0.720)	0.306 (0.240-0.382)	0.786 (0.735-0.830)
Senior radiologist 1	0.693 (0.616-0.761)	0.622 (0.461-0.759)	0.717 (0.628-0.792)	0.418 (0.297-0.550)	0.853 (0.768-0.910)
Senior radiologist 2	0.713 (0.636-0.780)	0.568 (0.409-0.713)	0.761 (0.675-0.830)	0.438 (0.307-0.577)	0.843 (0.760-0.901)
Senior radiologist 3	0.740 (0.664-0.804)	0.541 (0.384-0.690)	0.805 (0.723-0.868)	0.476 (0.334-0.623)	0.843 (0.762-0.899)
All senior radiologists	0.716 (0.672-0.755)	0.577 (0.484-0.664)	0.761 (0.713-0.803)	0.441 (0.363-0.523)	0.846 (0.801-0.882)
Radiologists assisted by CytoNet					
Junior radiologist 1	0.647 (0.567-0.719)	0.541 (0.384-0.690)	0.681 (0.591-0.760)	0.357 (0.245-0.488)	0.819 (0.729-0.884)
Junior radiologist 2	0.633 (0.554-0.706)	0.595 (0.435-0.737)	0.646 (0.554-0.728)	0.355 (0.247-0.479)	0.830 (0.738-0.894)
Junior radiologist 3	0.620 (0.540-0.694)	0.541 (0.384-0.690)	0.646 (0.554-0.728)	0.333 (0.227-0.459)	0.811 (0.718-0.879)
All junior radiologists	0.633 (0.588-0.677)	0.559 (0.466-0.647)	0.658 (0.606-0.706)	0.348 (0.282-0.421)	0.820 (0.770-0.861)
Senior radiologist 1	0.707 (0.629-0.774)	0.676 (0.515-0.804)	0.717 (0.628-0.792)	0.439 (0.318-0.567)	0.871 (0.788-0.925)
Senior radiologist 2	0.727 (0.650-0.792)	0.622 (0.461-0.759)	0.761 (0.675-0.830)	0.460 (0.330-0.596)	0.860 (0.779-0.915)
Senior radiologist 3	0.760 (0.686-0.821)	0.649 (0.488-0.782)	0.796 (0.713-0.860)	0.511 (0.372-0.647)	0.874 (0.796-0.925)
All senior radiologists	0.731 (0.688-0.770)	0.649 (0.556-0.731)	0.758 (0.710-0.801)	0.468 (0.390-0.546)	0.868 (0.825-0.902)

CytoNet, Cytology-based multitask predictive Network.

Supplementary Table 7. Comparison of CytoNet and the single-task model for disease-free survival prediction in bladder cancer

Models	C index (95% CI)			
	TC	IVC	EVC1	EVC2
CytoNet	0.710 (0.669-0.751)	0.693 (0.635-0.750)	0.680 (0.619-0.742)	0.689 (0.591-0.786)
Single-task model	0.655 (0.612-0.697)	0.638 (0.575-0.702)	0.633 (0.575-0.691)	0.622 (0.514-0.731)

CytoNet, Cytology-based multitask predictive Network; C index, concordance index; TC, training cohort; IVC, internal validation cohort; EVC1, external validation cohort 1; EVC2, external validation cohort 2.

Supplementary Table 8. Assessment of model performance for disease-free survival prediction in bladder cancer

Models	C index (95% CI)			
	TC	IVC	EVC1	EVC2
Clinical COX	0.683 (0.625-0.712)	0.669 (0.628-0.712)	0.712 (0.627-0.711)	0.643 (0.624-0.712)
CytoNet	0.710 (0.669-0.751)	0.693 (0.635-0.750)	0.680 (0.619-0.742)	0.689 (0.591-0.786)
Integrated COX	0.781 (0.698-0.771)	0.750 (0.698-0.770)	0.750 (0.698-0.769)	0.730 (0.697-0.771)

CytoNet, Cytology-based multitask predictive Network; C index, concordance index; TC, training cohort; IVC, internal validation cohort; EVC1, external validation cohort 1; EVC2, external validation cohort 2.

Supplementary Table 9. Performance of the CytoNet and radiology in predicting lymph node metastasis in the independent external test cohort

	AUC (95% CI)	Accuracy (95% CI)	Sensitivity (95% CI)	Specificity (95% CI)	Positive predictive value (95% CI)	Negative predictive value (95% CI)
CytoNet	0.833 (0.742-0.924)	0.814 (0.727-0.877)	0.783 (0.581-0.903)	0.823 (0.724-0.891)	0.562 (0.393-0.718)	0.929 (0.843-0.969)
Radiology	-	0.676 (0.581-0.759)	0.609 (0.408-0.778)	0.696 (0.588-0.787)	0.368 (0.234-0.527)	0.859 (0.754-0.924)

CytoNet, Cytology-based multitask predictive Network; AUC, area under the curve.

Supplementary Table 10. Performance of radiology in predicting lymph node metastasis

	ACC (95% CI)	SEN (95% CI)	SPE (95% CI)	PPV (95% CI)	NPV (95% CI)
**Training cohort**					
CT	0.701 (0.635-0.760)	0.367 (0.247-0.507)	0.806 (0.737-0.861)	0.375 (0.252-0.516)	0.801 (0.732-0.856)
MRI	0.723 (0.667-0.772)	0.455 (0.330-0.585)	0.790 (0.731-0.839)	0.352 (0.251-0.468)	0.852 (0.797-0.894)
**Internal validation cohort**					
CT	0.631 (0.538-0.715)	0.500 (0.326-0.674)	0.675 (0.568-0.766)	0.341 (0.216-0.494)	0.800 (0.692-0.877)
MRI	0.809 (0.718-0.875)	0.353 (0.173-0.587)	0.909 (0.824-0.955)	0.462 (0.232-0.709)	0.864 (0.773-0.922)
**External validation cohort 1**					
CT	0.505 (0.411-0.599)	0.524 (0.324-0.717)	0.500 (0.395-0.605)	0.208 (0.120-0.335)	0.808 (0.681-0.892)
MRI	0.766 (0.697-0.823)	0.806 (0.650-0.902)	0.756 (0.677-0.820)	0.468 (0.349-0.590)	0.936 (0.873-0.969)
**External validation cohort 2**					
CT	0.548 (0.378-0.708)	0.600 (0.231-0.882)	0.538 (0.355-0712)	0.200 (0.070-0.452)	0.875 (0.640-0.965)
MRI	0.762 (0.615-0.865)	0.667 (0.354-0.879)	0.788 (0.623-0.893)	0.462 (0.232-0.709)	0.897 (0.736-0.964)
**Bladder overall cohort**					
CT	0.627 (0.582-0.671)	0.447 (0.354-0.543)	0.681 (0.630-0.728)	0.293 (0.227-0.368)	0.806 (0.757-0.847)
MRI	0.752 (0.716-0.785)	0.564 (0.474-0.650)	0.800 (0.761-0.834)	0.415 (0.341-0.493)	0.879 (0.845-0.907)
**Cervical training cohort**					
CT	0.540 (0.457-0.620)	0.441 (0.289-0.606)	0.571 (0.476-0.662)	0.250 (0.158-0.372)	0.759 (0.655-0.840)
MRI	0.715 (0.660-0.764)	0.525 (0.400-0.647)	0.764 (0.705-0.815)	0.369 (0.274-0.476)	0.860 (0.805-0.901)
**Cervical validation cohort**					
CT	0.558 (0.423-0.684)	0.625 (0.386-0.815)	0.528 (0.370-0.680)	0.370 (0.215-0.558)	0.760 (0.566-0.885)
MRI	0.691 (0.605-0.766)	0.478 (0.292-0.670)	0.740 (0.646-0.816)	0.297 (0.175-0.458)	0.860 (0.772-0.918)
**Cervical overall cohort**					
CT	0.545 (0.474-0.614)	0.500 (0.366-0.634)	0.560 (0.478-0.639)	0.287 (0.203-0.390)	0.760 (0.669-0.832)
MRI	0.708 (0.662-0.750)	0.512 (0.406-0.617)	0.757 (0.708-0.800)	0.347 (0.268-0.436)	0.860 (0.815-0.896)
**Thyroid training cohort**					
Ultrasound	0.593 (0.571-0.615)	0.400 (0.370-0.431)	0.794 (0.767-0.819)	0.670 (0.630-0.706)	0.559 (0.532-0.586)
CT	0.633 (0.593-0.671)	0.613 (0.560-0.664)	0.658 (0.598-0.713)	0.695 (0.640-0.745)	0.572 (0.515-0.627)
**Thyroid validation cohort**					
Ultrasound	0.586 (0.552-0.620)	0.414 (0.368-0.462)	0.773 (0.728-0.812)	0.663 (0.604-0.718)	0.550 (0.508-0.591)
CT	0.640 (0.575-0.700)	0.615 (0.531-0.693)	0.678 (0.574-0.767)	0.748 (0.660-0.819)	0.532 (0.439-0.622)
**Thyroid overall cohort**					
Ultrasound	0.591 (0.572-0.609)	0.404 (0.379-0.430)	0.788 (0.765-0.809)	0.667 (0.635-0.698)	0.556 (0.534-0.579)
CT	0.635 (0.601-0.667)	0.614 (0.569-0.657)	0.663 (0.612-0.711)	0.710 (0.663-0.752)	0.561 (0.513-0.608)

ACC, accuracy; SEN, sensitivity; SPE, specificity; PPV, positive predictive value; NPV, negative predictive value; CT, computed tomography; MRI, magnetic resonance imaging.

Supplementary Table 11. Comparison of CytoNet with representative AI-based models for lymph node metastasis prediction in bladder cancer

Study	Input modality	Target task	Sample size	LNM performance (validation set)	Prognostic analysis	AI-assisted reader study	Pan-cancer validation
Wu et al., 2017[1]	CT images	LNM prediction	118 patients	AUC: 0.899	×	×	×
Wu et al., 2018[2]	MRI images	LNM prediction	103 patients	AUC: 0.845	×	×	×
Harmon et al., 2020[3]	Histopathological WSIs	LNM prediction	307 patients	AUC: 0.784	×	×	×
Gresser et al., 2023[4]	CT images	LNM prediction	391 patients	AUC: 0.810	×	×	×
Girard et al., 2023[5]	[18F]FDG PET/CT criteria	LNM prediction	173 patients	AUC: 0.590	×	×	×
Zheng et al., 2023[6]	Histopathological WSIs	LNM prediction	540 patients	AUC: 0.762	×	×	×
Ji et al., 2024[7]	Clinical variables	LNM prediction	655 patients	AUC: 0.855	×	×	×
Sun et al., 2024[8]	CT images	LNM prediction	239 patients	AUC: 0.834	√	×	×
CytoNet,	Cytology WSIs and clinical variables	Multitask LNM and DFS prediction	1134 patients	AUC: 0.890	√	√	√

CytoNet, Cytology-based multitask predictive Network; CT, computed tomography; MRI, magnetic resonance imaging; WSI, whole slide image; LNM, lymph node metastasis; DFS, disease-free survival; AUC, area under the curve; AI, artificial intelligence.

References

1. S. Wu, J. Zheng, Y. Li, et al., "A Radiomics Nomogram for the Preoperative Prediction of Lymph Node Metastasis in Bladder Cancer," *Clinical Cancer Research 23*, no. 22 (2017): 6904–6911, https://doi.org/10.1158/1078-0432.CCR-17-1510.

2. S. Wu, J. Zheng, Y. Li, et al., "Development and Validation of an MRI-Based Radiomics Signature for the Preoperative Prediction of Lymph Node Metastasis in Bladder Cancer," *EBioMedicine 34* (2018): 76–84, https://doi.org/10.1016/j.ebiom.2018.07.029.

3. S. A. Harmon, T. H. Sanford, G. T. Brown, et al., "Multiresolution Application of Artificial Intelligence in Digital Pathology for Prediction of Positive Lymph Nodes From Primary Tumors in Bladder Cancer," *JCO Clinical Cancer Informatics* no. 4 (2020): 367–382, https://doi.org/10.1200/CCI.19.00155.

4. E. Gresser, P. Woźnicki, K. Messmer, et al., "Radiomics Signature Using Manual Versus Automated Segmentation for Lymph Node Staging of Bladder Cancer," *European Urology Focus 9*, no. 1 (2023): 145–153, https://doi.org/10.1016/j.euf.2022.08.015.

5. A. Girard, L. Dercle, H. Vila-Reyes, et al., "A Machine-Learning-Based Combination of Criteria to Detect Bladder Cancer Lymph Node Metastasis on [18F]FDG PET/CT: A Pathology-

Controlled Study," *European Radiology 33*, no. 4 (2022): 2821–2829, https://doi.org/10.1007/s00330-022-09270-9.

6. Q. Zheng, J. Jian, J. Wang, et al., "Predicting Lymph Node Metastasis Status from Primary Muscle-Invasive Bladder Cancer Histology Slides Using Deep Learning: A Retrospective Multicenter Study," *Cancers 15*, no. 11 (2023): 3000, https://doi.org/10.3390/cancers15113000.

7. J. Ji, T. Zhang, L. Zhu, et al., "Using Machine Learning to Develop Preoperative Model for Lymph Node Metastasis in Patients with Bladder Urothelial Carcinoma," *BMC Cancer 24*, no. 1 (2024): 725, https://doi.org/10.1186/s12885-024-12467-4.

8. R. Sun, M. Zhang, L. Yang, et al., "Preoperative CT-Based Deep Learning Radiomics Model to Predict Lymph Node Metastasis and Patient Prognosis in Bladder Cancer: A Two-Center Study," *Insights into Imaging 15*, no. 1 (2024): 21, https://doi.org/10.1186/s13244-023-01569-5.

Supplementary Figures

Supplementary Figure 1

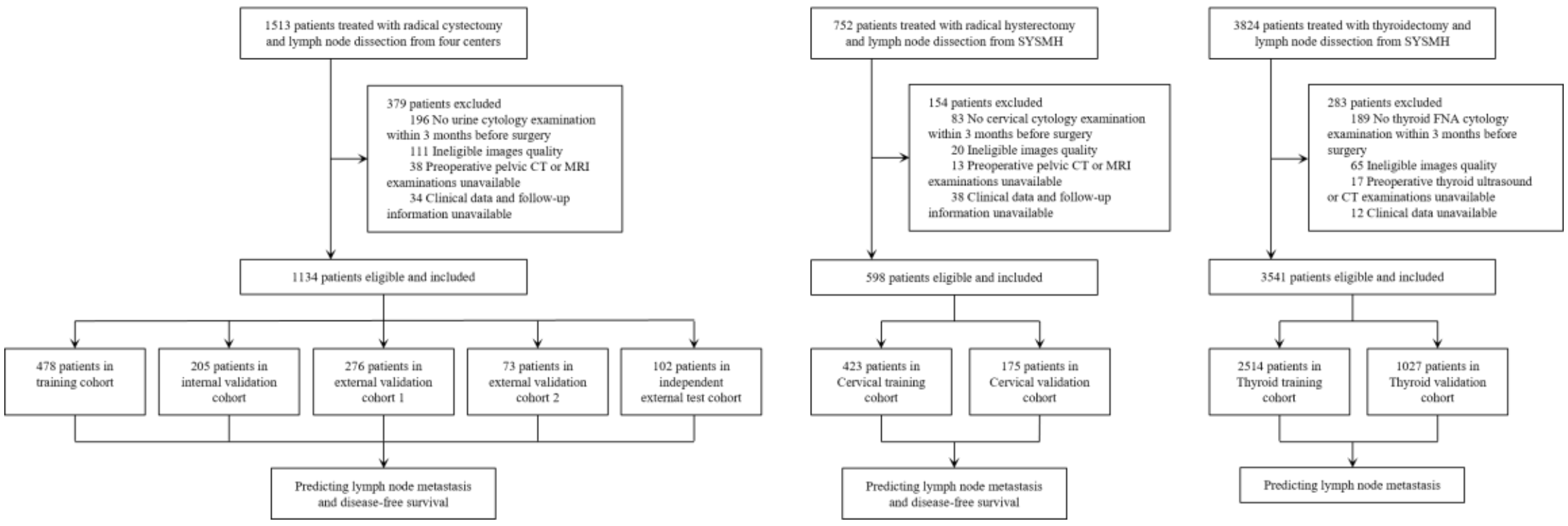

Supplementary Figure 1. Flowchart of patient selection and study cohort allocation

This flow diagram outlines the retrospective patient selection and cohort construction process across three cancer types (bladder, cervical, and thyroid) for developing and validating the CytoNet. CytoNet, Cytology-based multitask predictive Network; CT, computed tomography; MRI, magnetic resonance imaging; SYSMH, Sun Yat-sen Memorial Hospital of Sun Yat-sen University.

Supplementary Figure 2

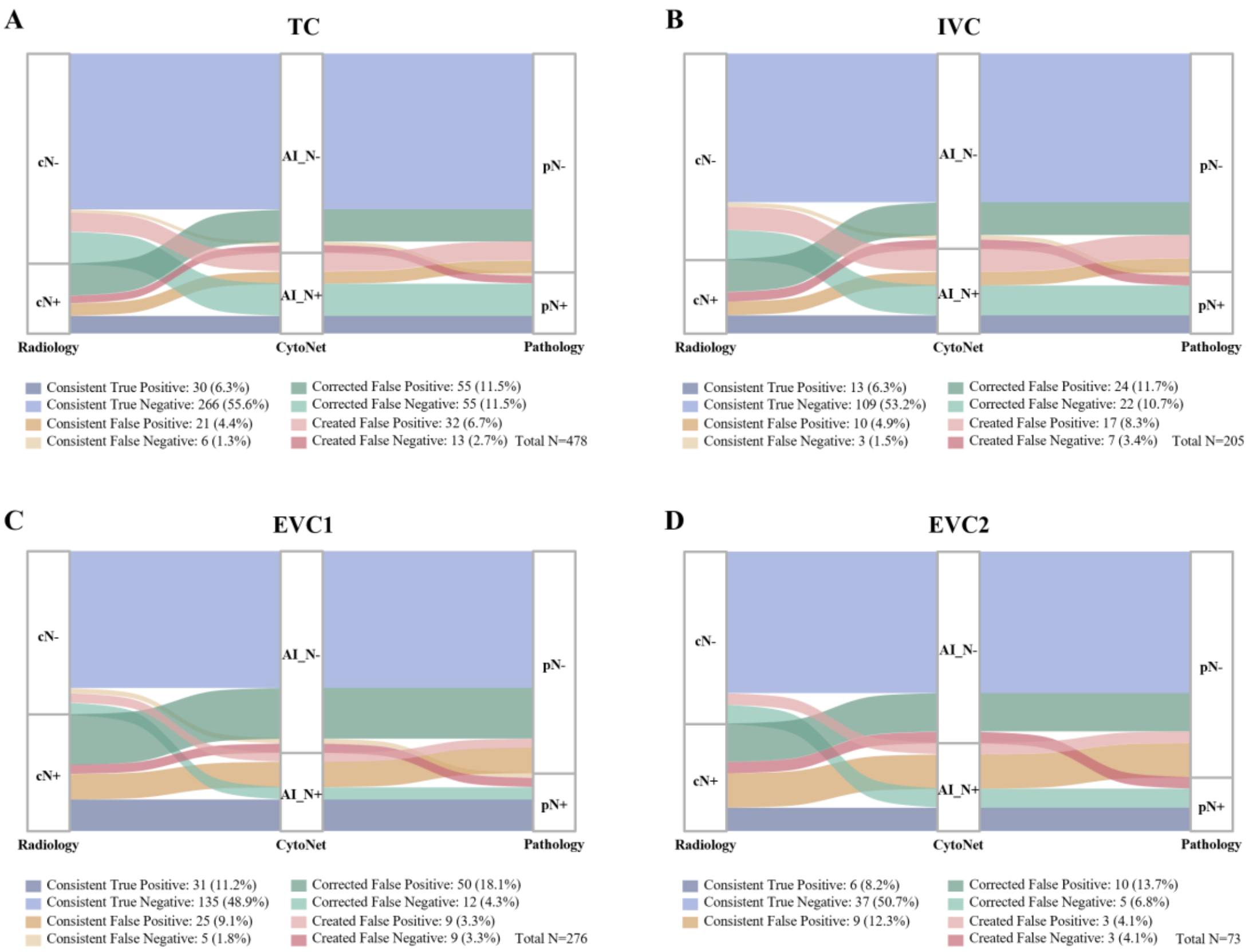

Supplementary Figure 2. Sankey diagrams visualize the concordance among radiology assessment, CytoNet prediction, and pathological diagnosis in different bladder cancer cohorts

(A-D) Sankey diagrams for the TC, IVC, EVC1, and EVC2, respectively. The left, middle, and right columns represent radiology-based nodal assessment, CytoNet-predicted nodal status, and pathological reference status, respectively. cN-/cN+ indicate radiology-based negative or positive lymph node status; AI_N-/AI_N+ indicate CytoNet-predicted negative or positive lymph node status; and pN-/pN+ indicate pathologically negative or positive lymph node status. The width of each flow is proportional to the number of patients. “Consistent” indicates that radiology and CytoNet gave the same classification relative to pathology; “corrected” indicates that CytoNet corrected a radiology misclassification; and “created” indicates that CytoNet introduced a new misclassification relative to radiology. CytoNet, Cytology-based

multitask predictive Network; TC, training cohort; IVC, internal validation cohort; EVC1, external validation cohort 1; EVC2, external validation cohort 2.

Supplementary Figure 3

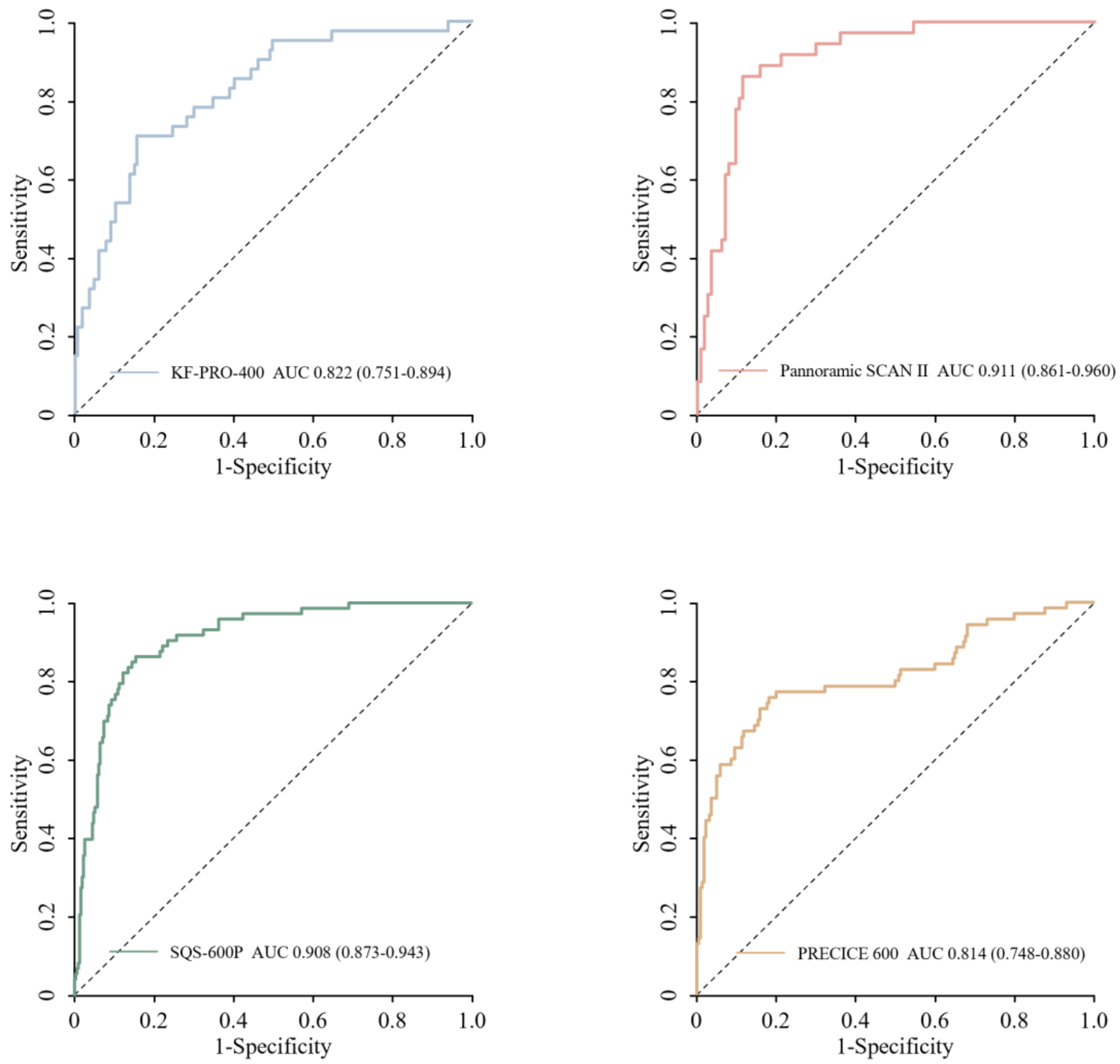

Supplementary Figure 3. Performance of the CytoNet for lymph node metastasis prediction in the combined cohort

Receiver operating characteristic curves evaluate the predictive performance of the CytoNet when applied to whole slide images acquired from four types of digital scanners. CytoNet, Cytology-based multitask predictive Network; AUC, area under the curve.

Supplementary Figure 4

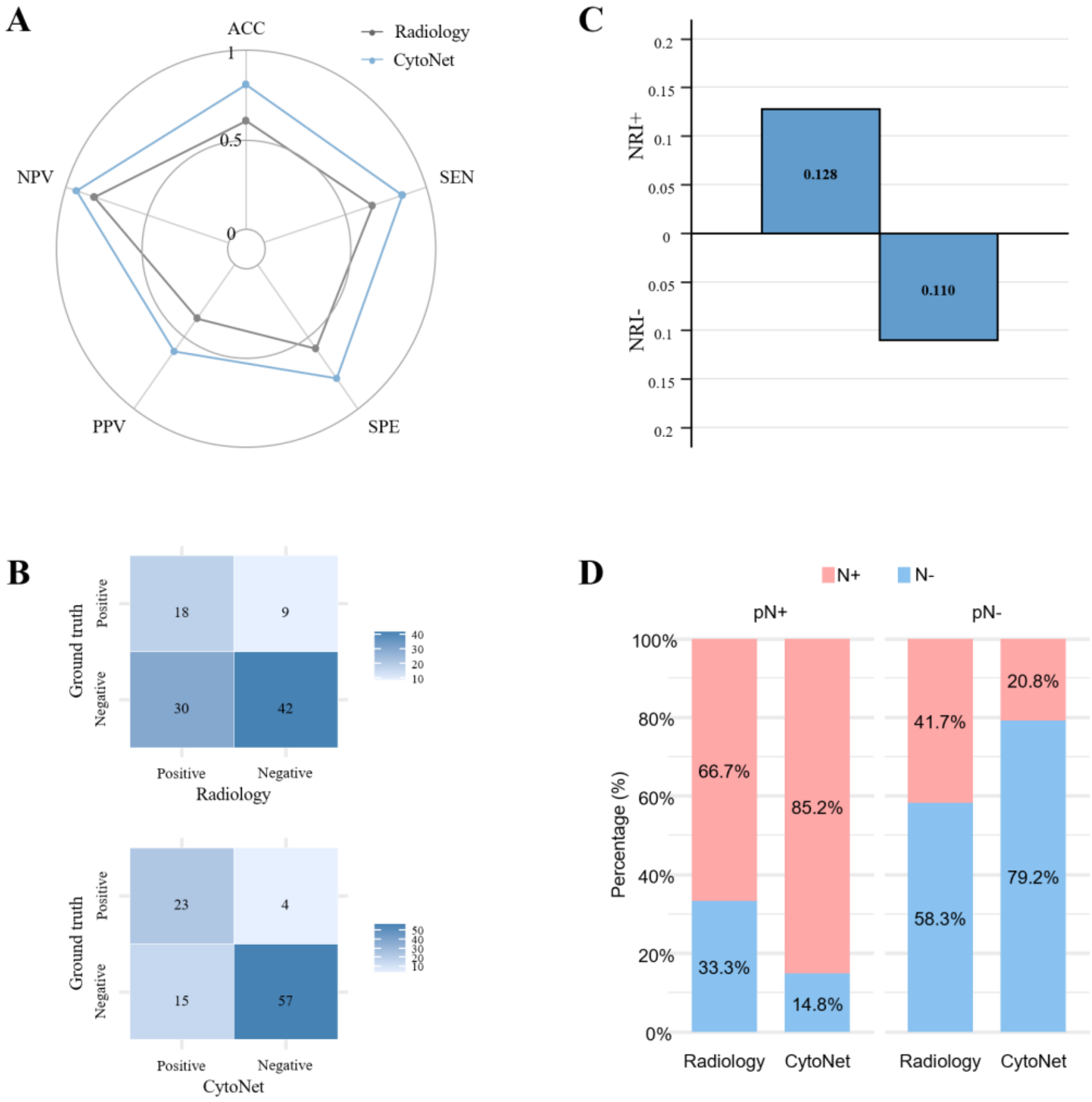

Supplementary Figure 4. Performance of CytoNet versus radiology for lymph node metastasis detection after neoadjuvant therapy

(A) Radar chart comparing the performance of radiology and CytoNet across five key diagnostic metrics: accuracy (ACC), sensitivity (SEN), specificity (SPE), positive predictive value (PPV), and negative predictive value (NPV). (B) Confusion matrices evaluating the diagnostic agreement of radiology (top) and CytoNet (bottom) with the pathological ground truth. (C) Net reclassification improvement (NRI) analysis quantifying the predictive gain of CytoNet over radiology. (D) Bar charts compare the predictive performance of radiology and CytoNet across different lymph node statuses. CytoNet, Cytology-based multitask predictive Network.

Supplementary Figure 5

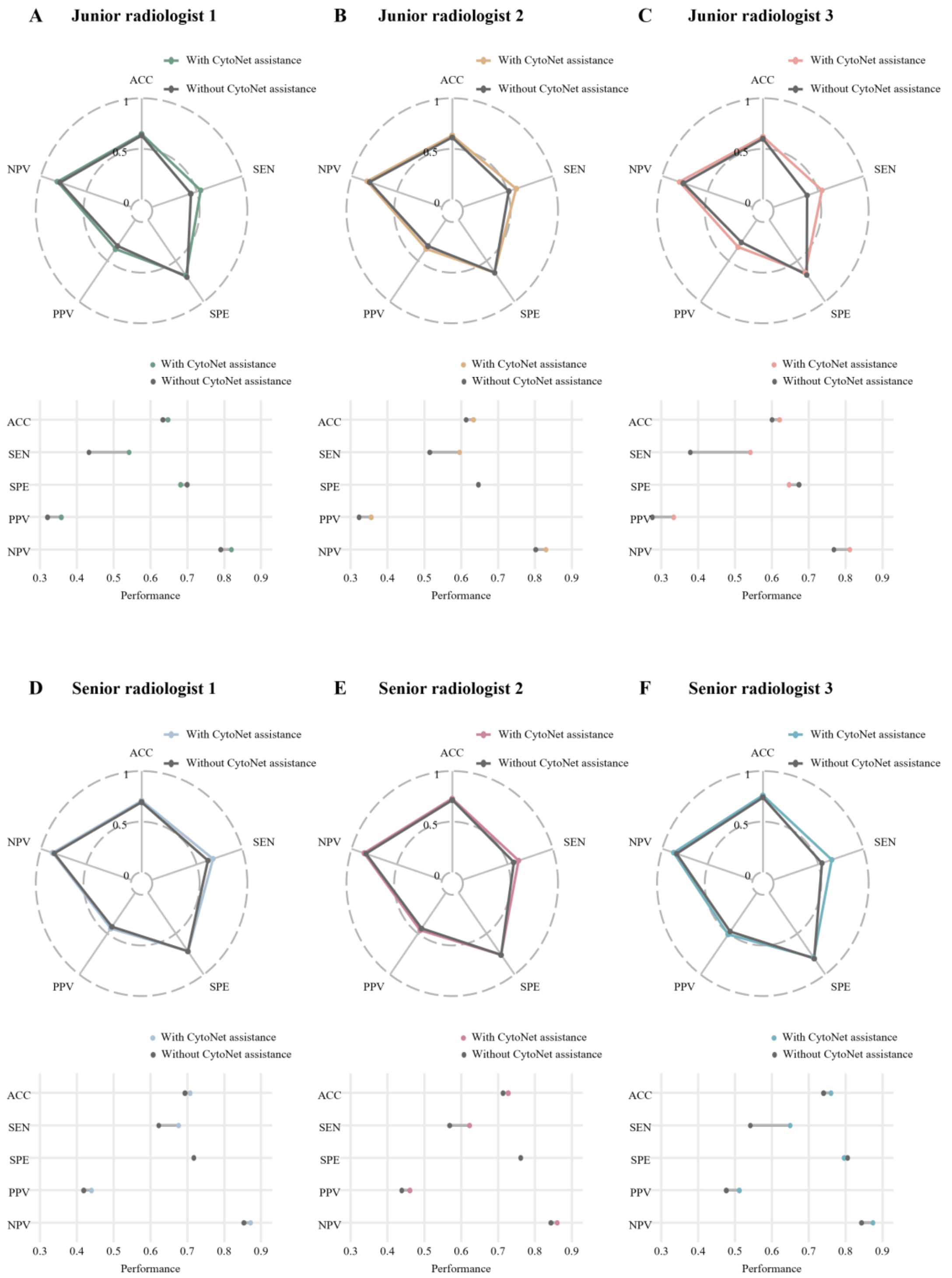

Supplementary Figure 5. Performance changes of radiologists with CytoNet assistance for preoperative lymph node metastasis prediction

(A-C) Radar charts and paired performance plots comparing the diagnostic performance of three junior radiologists with and without CytoNet assistance for

preoperative lymph node metastasis prediction. (D-F) Radar charts and paired performance plots comparing the diagnostic performance of three senior radiologists with and without CytoNet assistance. Radiologists first assessed LNM status using routine clinical information and CT or MRI images alone. After a washout period, they reassessed the same cases with additional access to cytology images, CAM heatmaps, and CytoNet-derived LNM-predicted risk scores. Five diagnostic metrics were evaluated: accuracy (ACC), sensitivity (SEN), specificity (SPE), positive predictive value (PPV), and negative predictive value (NPV). CytoNet, Cytology-based multitask predictive Network; LNM, lymph node metastasis; CAM, class activation mapping.

Supplementary Figure 6

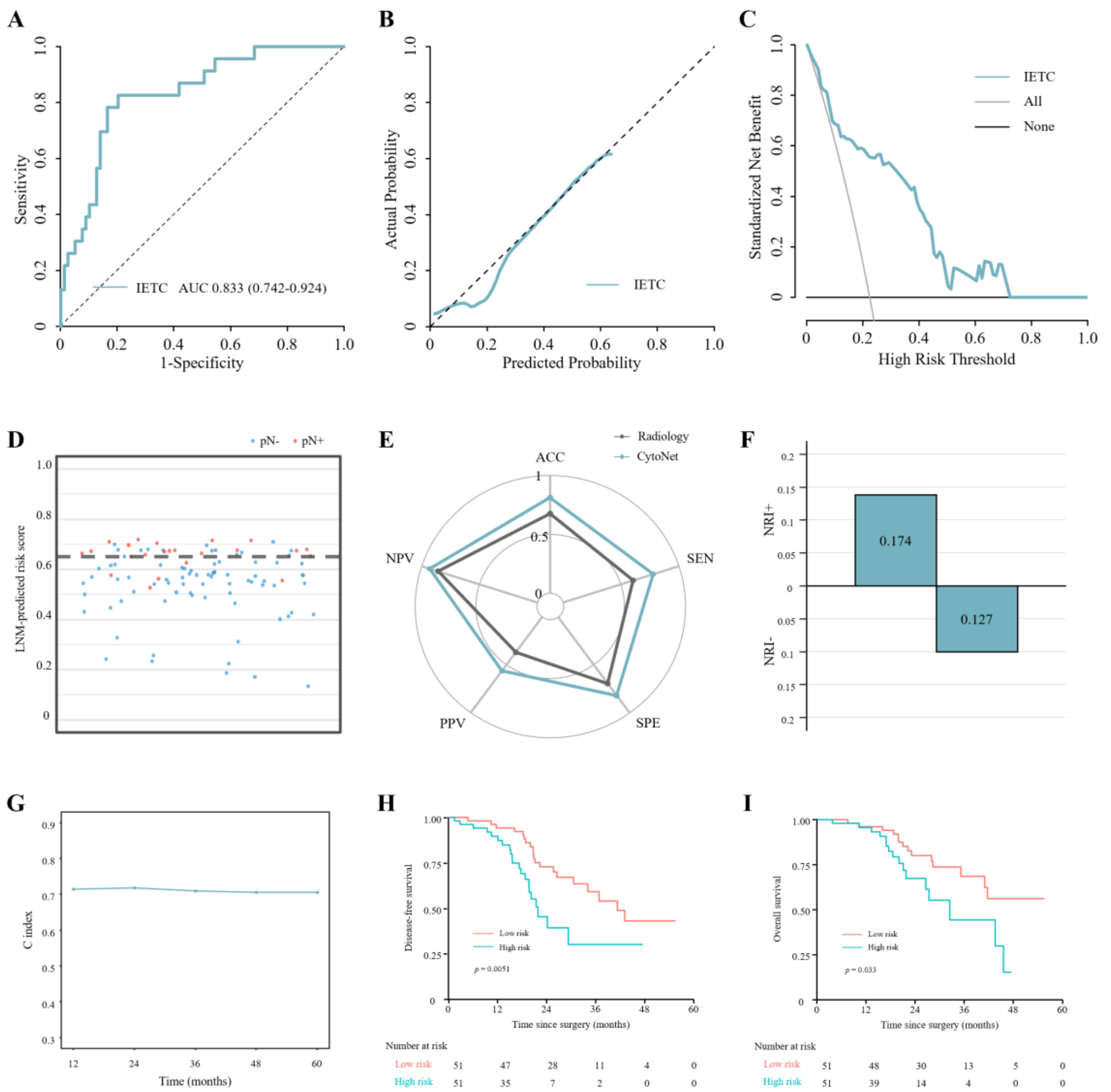

Supplementary Figure 6. Independent external testing of CytoNet in non-Sun Yat-sen University-affiliated hospitals

(A) Receiver operating characteristic curve of CytoNet for lymph node metastasis prediction in the independent external test cohort. (B) Calibration curve showing the agreement between predicted and observed probabilities. (C) Decision curve analysis evaluating the clinical net benefit of CytoNet across different threshold probabilities. (D) Scatter plot showing the distribution of LNM-predicted risk scores according to pathological lymph node status. (E) Radar chart comparing the diagnostic performance of CytoNet and radiological assessment across five key metrics. (F) Net reclassification improvement analysis quantifying the predictive gain of CytoNet over radiological assessment. (G) Time-dependent C indices of CytoNet for disease-free survival prediction. (H-I) Kaplan-Meier curves of disease-free survival and overall survival for patients stratified into low-risk and high-risk groups by CytoNet. *P* values were calculated using the log-rank test. CytoNet, Cytology-based multitask predictive Network; IETC, independent external test cohort; LNM, lymph node metastasis; AUC, area under the curve; ACC, accuracy; SEN, sensitivity; SPE, specificity; PPV, positive predictive value; NPV, negative predictive value; NRI, net reclassification improvement; pN-, pathologically negative lymph nodes; pN+, pathologically positive lymph nodes; C index, concordance index.

Supplementary Figure 7

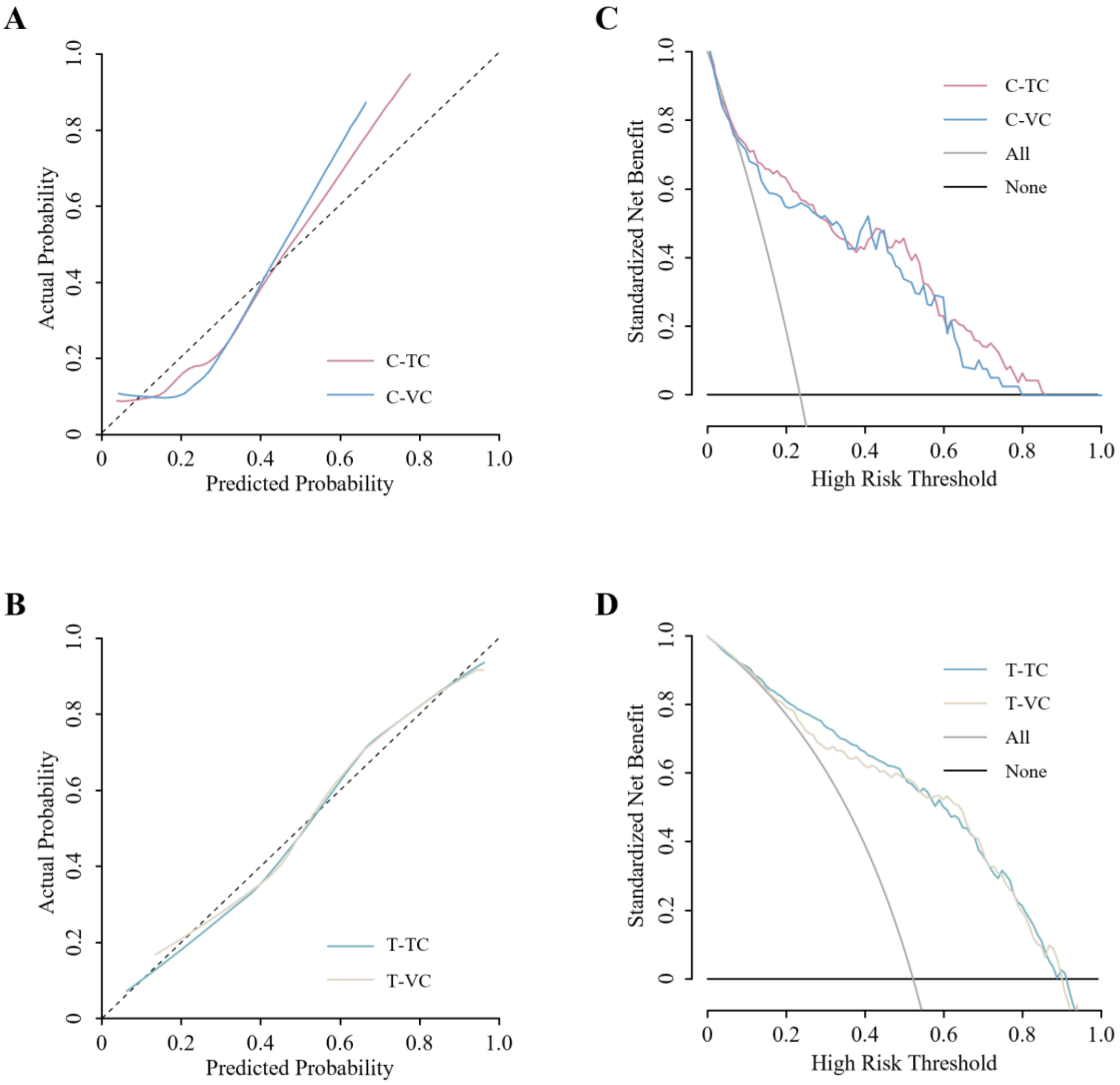

Supplementary Figure 7. Model performance for predicting lymph node metastasis in cervical and thyroid cancers

Calibration curves (A-B) and decision curve analysis curves (C-D) evaluate the predictive accuracy and clinical utility of CytoNet across cervical and thyroid cancer cohorts. CytoNet, Cytology-based multitask predictive Network; C-TC, cervical training cohort; C-VC, cervical validation cohort; T-TC, thyroid training cohort; T-VC, thyroid validation cohort.

Supplementary Figure 8

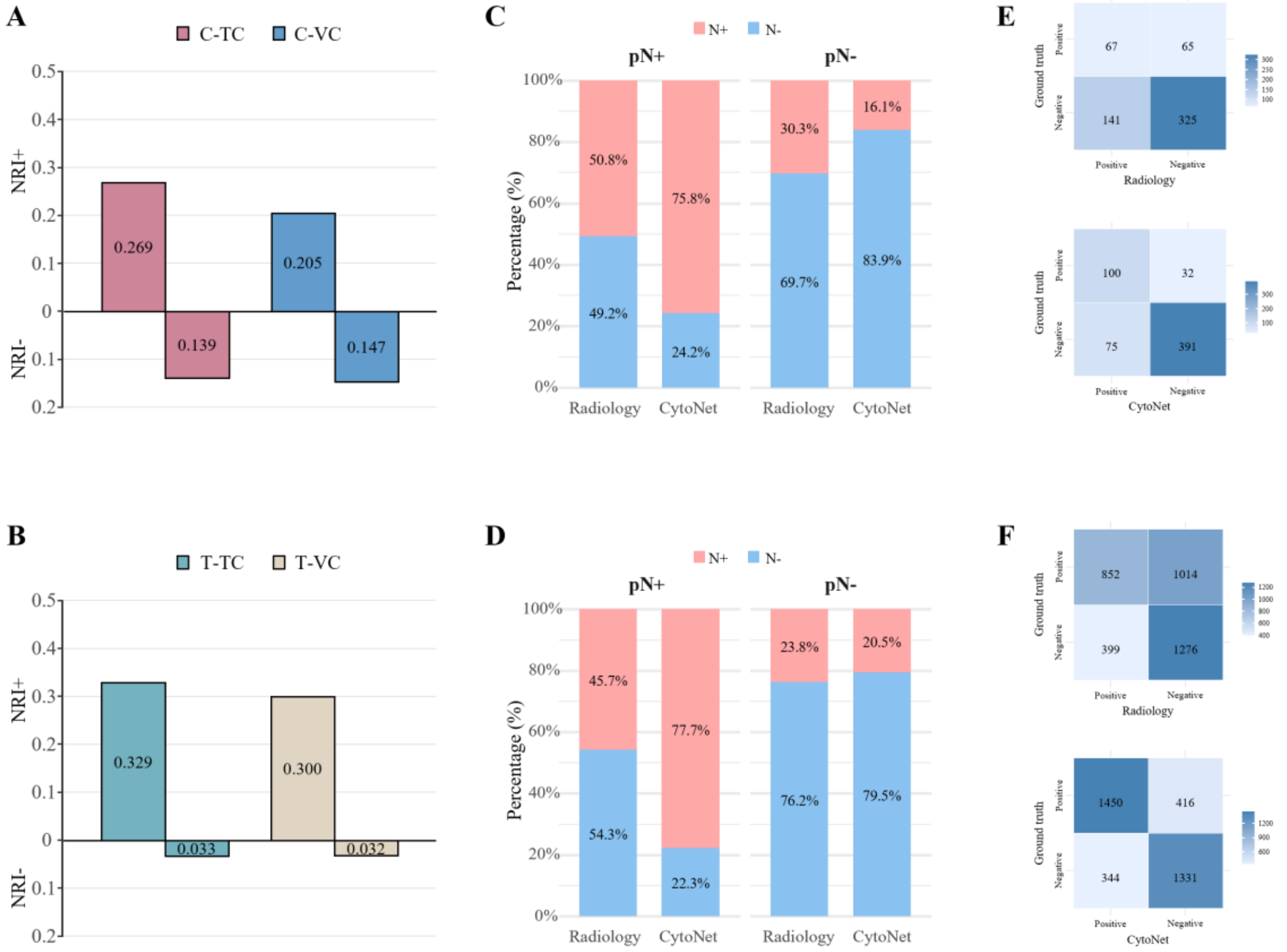

Supplementary Figure 8. Comprehensive performance comparison between radiology and the CytoNet for lymph node metastasis prediction in cervical and thyroid cancers

(A-B) Net reclassification improvement analyses show the performance change in predicting lymph node metastasis of the CtyoMet compared with radiology. (C-D) Bar charts compare the predictive performance of radiology and CytoNet across different lymph node statuses. (E-F) Confusion matrices compare the performance of the CytoNet and radiology. CytoNet, Cytology-based multitask predictive Network; NRI, net reclassification improvement; C-TC, cervical training cohort; C-VC, cervical validation cohort; T-TC, thyroid training cohort; T-VC, thyroid validation cohort.

Supplementary Figure 9

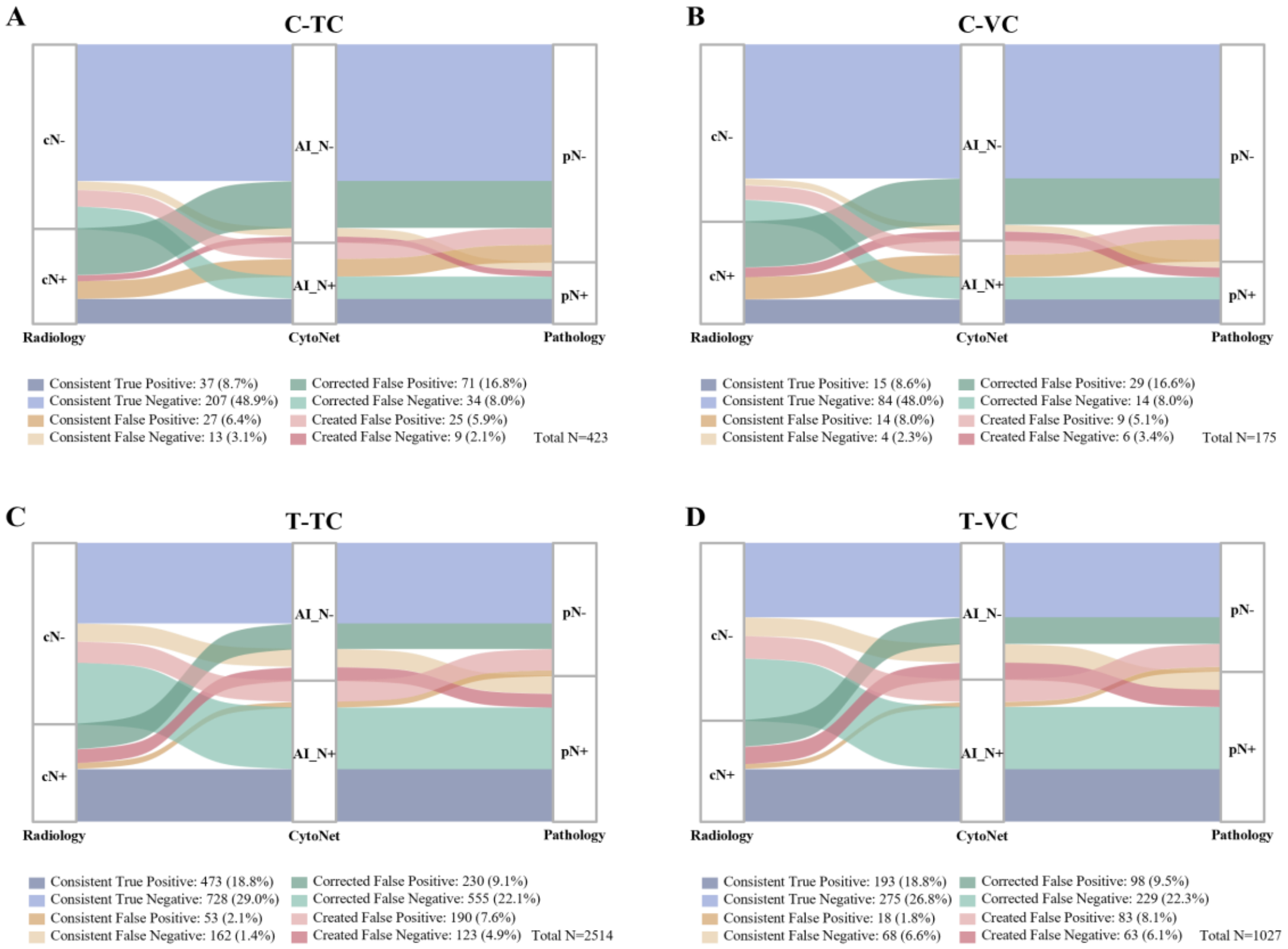

Supplementary Figure 9. Sankey diagrams visualize the concordance among radiology assessment, CytoNet prediction, and pathological diagnosis across cervical and thyroid cancer cohorts

(A-D) Sankey diagrams for the C-TC, C-VC, T-TC, and T-VC, respectively. The left, middle, and right columns represent radiology-based nodal assessment, CytoNet-predicted nodal status, and pathological reference status, respectively. cN-/cN+ indicate radiology-based negative or positive lymph node status; AI_N-/AI_N+ indicate CytoNet-predicted negative or positive lymph node status; and pN-/pN+ indicate pathologically negative or positive lymph node status. The width of each flow is proportional to the number of patients. “Consistent” indicates that radiology and CytoNet gave the same classification relative to pathology; “corrected” indicates that CytoNet corrected a radiology misclassification; and “created” indicates that CytoNet introduced a new misclassification relative to radiology. CytoNet, Cytology-based multitask predictive Network; C-TC, cervical training cohort; C-VC, cervical validation cohort; T-TC, thyroid training cohort; T-VC, thyroid validation cohort.
